# CBGA ameliorates inflammation and fibrosis in nephropathy

**DOI:** 10.1038/s41598-023-33507-2

**Published:** 2023-04-18

**Authors:** Sayuri Suzuki, Andrea Fleig, Reinhold Penner

**Affiliations:** 1 Center for Biomedical Research, The Queen’s Medical Center, 1301 Punchbowl St., Honolulu, HI 96813 USA; 2 University of Hawaii Cancer Center, 651 Ilalo St., Honolulu, HI 96813 USA; 3 John A. Burns School of Medicine, University of Hawaii, 651 Ilalo St., Honolulu, HI 96813 USA

**Keywords:** Drug discovery, Kidney, Kidney diseases

## Abstract

Cannabidiol (CBD) is thought to have multiple biological effects, including the ability to attenuate inflammatory processes. Cannabigerols (CBGA and its decarboxylated CBG molecule) have pharmacological profiles similar to CBD. The endocannabinoid system has recently emerged to contribute to kidney disease, however, the therapeutic properties of cannabinoids in kidney disease remain largely unknown. In this study, we determined whether CBD and CBGA can attenuate kidney damage in an acute kidney disease model induced by the chemotherapeutic cisplatin. In addition, we evaluated the anti-fibrosis effects of these cannabinoids in a chronic kidney disease model induced by unilateral ureteral obstruction (UUO). We find that CBGA, but not CBD, protects the kidney from cisplatin-induced nephrotoxicity. CBGA also strongly suppressed mRNA of inflammatory cytokines in cisplatin-induced nephropathy, whereas CBD treatment was only partially effective. Furthermore, both CBGA and CBD treatment significantly reduced apoptosis through inhibition of caspase-3 activity. In UUO kidneys, both CBGA and CBD strongly reduced renal fibrosis. Finally, we find that CBGA, but not CBD, has a potent inhibitory effect on the channel-kinase TRPM7. We conclude that CBGA and CBD possess reno-protective properties, with CBGA having a higher efficacy, likely due to its dual anti-inflammatory and anti-fibrotic effects paired with TRPM7 inhibition.

## Introduction

Kidney diseases can be chronic (CKD), where the loss of kidney function occurs gradually over time, or develop more rapidly within hours or a few days, where acute kidney injury (AKI) may be caused by factors such as dehydration, infection, or medication toxicity. Kidney diseases are initiated and maintained by a complex interplay of various mechanisms, including inflammation, oxidative stress, epithelial-to-mesenchymal transition (EMT), activation of cytokines and growth factors, and genetic factors. Various rodent models for such kidney diseases exist and understanding the mechanisms involved is crucial in developing effective treatments for kidney disease.

Cisplatin is an anti-tumor drug that is used as a chemotherapeutic in various malignancies, however, it has significant side effects with strong potential to cause nephrotoxicity. In fact, 20–30% of patients undergoing chemotherapy with cisplatin develop acute kidney damage due to nephrotoxicity, and some patients experience irreversible renal failure^1^. The cisplatin-induced nephropathy mouse model is generally recognized as a simple and reproducible model with clinical relevance and has been widely used for research of acute kidney injury^2–4^. Cisplatin induces inflammation, oxidative stress, vascular and proximal tubular injury, and apoptotic or necrotic cell death, which all contribute to kidney tissue damage and decline of renal function^3^.

The Unilateral Ureteral Obstruction (UUO) model is widely used as a chronic kidney disease (CKD) model associated with progressive tubulointerstitial injury^5–7^. Obstruction of the ureter causes the infiltration of monocytes and macrophages into the renal tubular interstitium. Activated macrophages produce cytokines, including tumor necrosis factor-α (TNF-α) and transforming growth factor-β1 (TGF-β1), resulting in increased cell proliferation of renal tubular epithelial cell and interstitial cells. In the damaged kidney, cell proliferation is a central response to injury that culminates in the development of fibrosis and renal failure. Tubular epithelial cell proliferation leads to tubule dilation, the fracture of tubular membrane, renal tubule loss, and an increase in myofibroblasts (transformed fibroblasts expressing alpha smooth muscle actin (α-SMA)) via epithelial-to-mesenchymal transition (EMT)^5–8^. Myofibroblasts produce extracellular matrix (ECM) proteins such as collagen, fibronectin and vimentin. The ECM is deposited in the space generated by tubule loss, and the replacement of renal tubules by ECM leads to fibrosis. Thus, cell transformation, proliferation, and apoptosis result in progressive cell loss, renal tubular atrophy and interstitial fibrosis, all of which critically contribute to progressive renal damage and loss of function in kidney disease.

Cannabis has historically and anecdotally been used to treat numerous ailments including chronic pain, muscle spasms, anxiety, and nausea. It is also used to help with sleep and to improve appetite. In recent years, there has been a growing interest in using Cannabis to treat epilepsy^9–11^ and multiple sclerosis^12–16^. The two major classes of natural products found in Cannabis are cannabinoid and terpenoid compounds, both of which have been reported to possess anti-inflammatory and analgesic properties. Cannabinoids have multiple mechanisms of action and impinge on a variety of biological targets, including G protein-coupled receptors such as cannabinoid receptors type 1 and 2 (CB1 and CB2)^17^ and serotonin receptor 5-HT1A and 5-HT2A, as well as various ion channels (e.g., TRPV1-TRPV4, TRPA1, and TRPM8). Both Δ^9^-tetrahydrocannabinol (THC) and cannabidiol (CBD) bind to the CB1 receptor, but only THC will activate CB1 receptors and mediate psychoactive effects, whereas CBD is thought to counteract inflammatory processes through unknown mechanisms. In addition to targeting the endocannabinoid system, both Δ^9^-THC and CBD have multiple additional pharmacological effects through cannabinoid receptor-independent mechanisms^18,19^, but mechanistic understanding remains largely unknown due to a heavy regulatory burden on the Cannabis plant^20^.

A third cannabinoid, cannabigerol (CBG) has a pharmacological profile similar to THC and CBD. CBG has been reported to have neuroprotective properties in a mouse model of Huntington’s disease^21^. The acidic form of CBG (Cannabigerolic acid or CBGA) as well as quinone derivatives of CBG also have neuroprotective properties in a Parkinson’s disease model^22^. CBGA properties and pharmacological effects remain to be elucidated, but our recent observation that CBGA is the most potent cannabinoid inhibitor of Store-Operated Calcium Entry (SOCE) and IL-2 production in T cells makes it a promising candidate molecule to modulate calcium signaling and inflammatory mechanisms in a variety of pro-inflammatory immune cells, potentially affecting kidney inflammation^23^.

The endocannabinoid system has recently emerged as an important player in the pathogenesis of progressive chronic kidney disease, diabetic nephropathy and drug nephrotoxicity as CB receptors seem to contribute to kidney disease^24^ and promote kidney damage to a varying degree. CB1 activation promotes inflammation and kidney injury^25,26^, it also serves as a major mediator to promote fibrosis not only in kidney but also liver^27,28^. Conversely, CB2 activation may have anti-inflammatory and reno-protective effects^29,30^. In this study, we determined whether CBD and CBGA can attenuate kidney damage in both an acute kidney disease model induced by cisplatin and additionally evaluated anti-fibrotic effects of these cannabinoids in a chronic kidney disease model induced by unilateral ureteral obstruction.

## Results

### CBGA and CBD have protective effects in acute kidney injury

**Figure 1:**
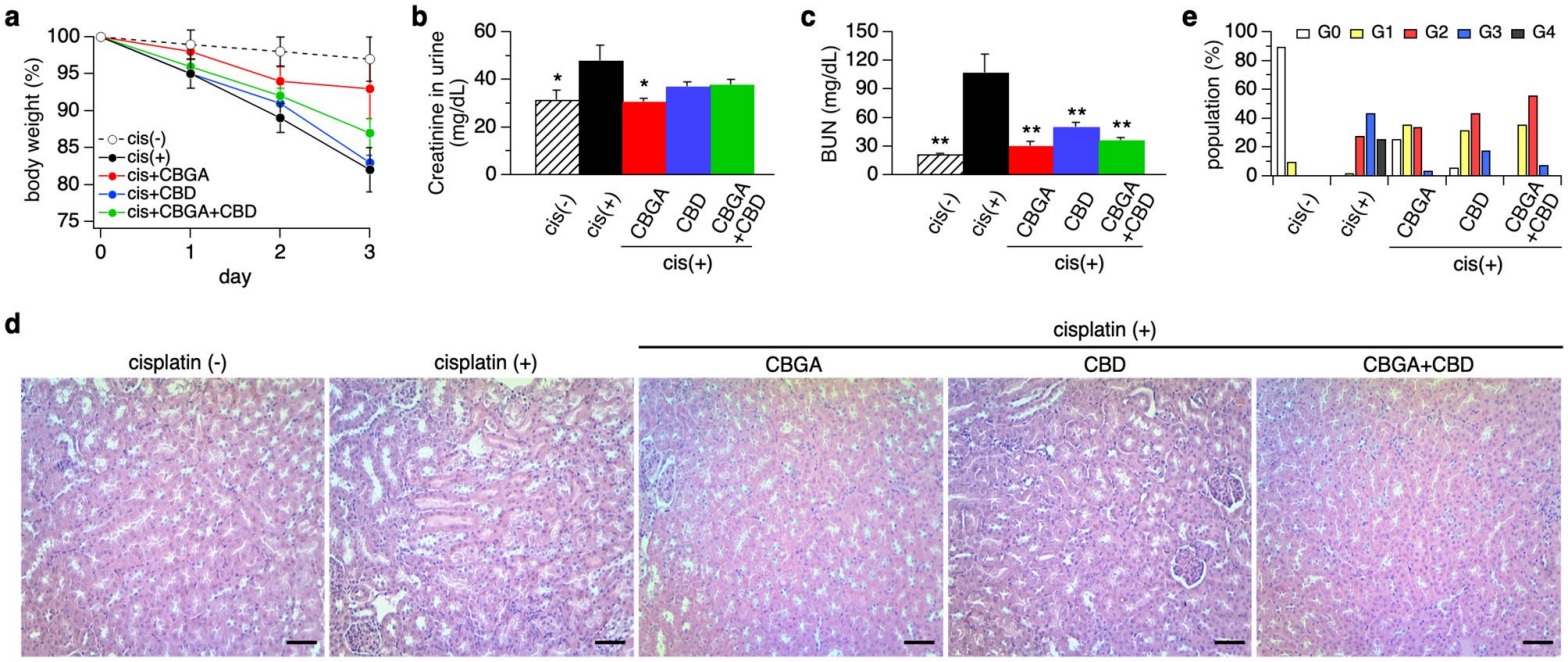
CBGA and CBD prevent kidney functional loss and damage in the cisplatin-induced acute nephropathy mouse model. (**a**) Body weight of mice was measured daily before administration of CBGA (10 mg/kg, red circles), CBD (10 mg/kg, blue circles), CBGA + CBD (each 10 mg/kg, green circles) from day 0 to day 3. The cisplatin (+) group was injected daily with vehicle in cisplatin-administered mice as a control (cis (+), black circles). Cisplatin was injected 2 h after the first cannabinoid administration at day 0. The animal group without cisplatin administration received vehicle instead of cisplatin as a negative control against cisplatin-induced nephropathy (cis (−), n = 4, white circles). Body weight of each day was normalized by the weight before cannabinoid injection at day 0 and the average is indicated (n = 5). (**b**) Creatinine levels were determined at day 3 in urine. (**c**) BUN levels were determined at day 3. (**d**) Representative pictures of HE staining taken from kidney sections in each treatment group (magnification × 200). Scale bars represent 50 µm. (**e**) The grading of tubular injury was quantified from HE staining and plotted as follows: G0 (grade 0, none, 0%, white bars); G1 (grade1, weak, ≤ 20%, yellow bars); G2 (grade 2, mild, > 20 to ≤ 50%, red bars); G3 (grade 3, moderate, > 50 to ≤ 80%, blue bars); G4 (grade 4, strong, > 80%, black bars). *p < 0.05, **p < 0.01 vs. cisplatin (+) group.

We assessed the effect of CBGA and CBD in acute kidney nephropathy induced by cisplatin administration (see “Materials and methods”). Cisplatin caused body weight loss of 18% in mice at day 3 after cisplatin administration (Fig. 1a; black circles and Table 1) compared to 3% of untreated mice as negative controls (Fig. 1a; white circles). CBGA treatment reduced body weight loss to 7% (Fig. 1a; red circles), whereas CBD treatment had no protective effect and body weight loss was 17% (Fig. 1a; blue circles). CBGA + CBD combination treatment partially reversed the protective effect on body weight loss seen with CBGA treatment to 13% (Fig. 1a; green circles), but still less than CBGA alone.

**Table 1:** Physiological parameters of cisplatin-induced mice at day 3.

	Body weight (g)	Kidney weight (mg)		Water intake (ml)	Urine (ml)
		Left	Right		
Cisplatin (−)	24.59 ± 1.03	172 ± 14.3	175 ± 16.7	4.65 ± 0.62	1.586 ± 0.137
Cisplatin (+)	18.36 ± 0.58**	116 ± 1.9**	117 ± 6.2**	1.14 ± 0.13**	0.513 ± 0.198**
Cis + CBGA	20.85 ± 1.29	128 ± 8.2**	139 ± 10.8	2.50 ± 0.50*	1.162 ± 0.128
Cis + CBD	20.23 ± 0.82*	121 ± 5.0**	132 ± 6.8	1.79 ± 0.37**	1.042 ± 0.131
Cis + CBGA + CBD	19.70 ± 0.66*	123 ± 7.3**	135 ± 8.1	1.14 ± 0.16**	0.877 ± 0.073*

Mice were sacrificed at day 3 after cisplatin administration and collected kidneys (n = 4–5). *p < 0.05, **p < 0.01 vs. cisplatin (−).

To evaluate kidney function under these experimental conditions, we collected urine and blood at day 3 and observed the expected increase of urine creatinine in mice treated with cisplatin (Fig. 1b; black bar). Mice treated with CBGA reversed this effect to levels observed in healthy mice without cisplatin treatment (Fig. 1b; red bar) and slightly less so by CBD and CBGA + CBD (Fig. 1b; blue and green bars). We next assessed blood urea nitrogen (BUN) levels as a measure of kidney function and found that they were significantly increased in cisplatin-induced nephropathic mice (Fig. 1c; black bar). This BUN increase was strongly suppressed in mice treated with CBGA, CBD and CBGA + CBD (Fig. 1c; red, blue and green bars).

Cisplatin administration also resulted in the loss of kidney weight compared to untreated mice, an effect that was alleviated by CBGA, CBD and CBGA + CBD treatment (Table 1). Histopathology revealed that cisplatin caused moderate to strong tubular necrosis and tubular dilation (cisplatin (+) in Fig. 1d,e) compared to healthy kidneys not exposed to cisplatin (cisplatin (−) in Fig. 1d,e). CBGA, CBD and CBGA + CBD treatment reduced this cisplatin-mediated kidney damage so that tubular injury levels were classified as weak to mild (Fig. 1d,e). We conclude that CBGA and CBD have a protective effect on cisplatin-induced acute kidney damage.

### CBGA ameliorates inflammation in cisplatin-induced acute nephropathy

**Figure 2:**
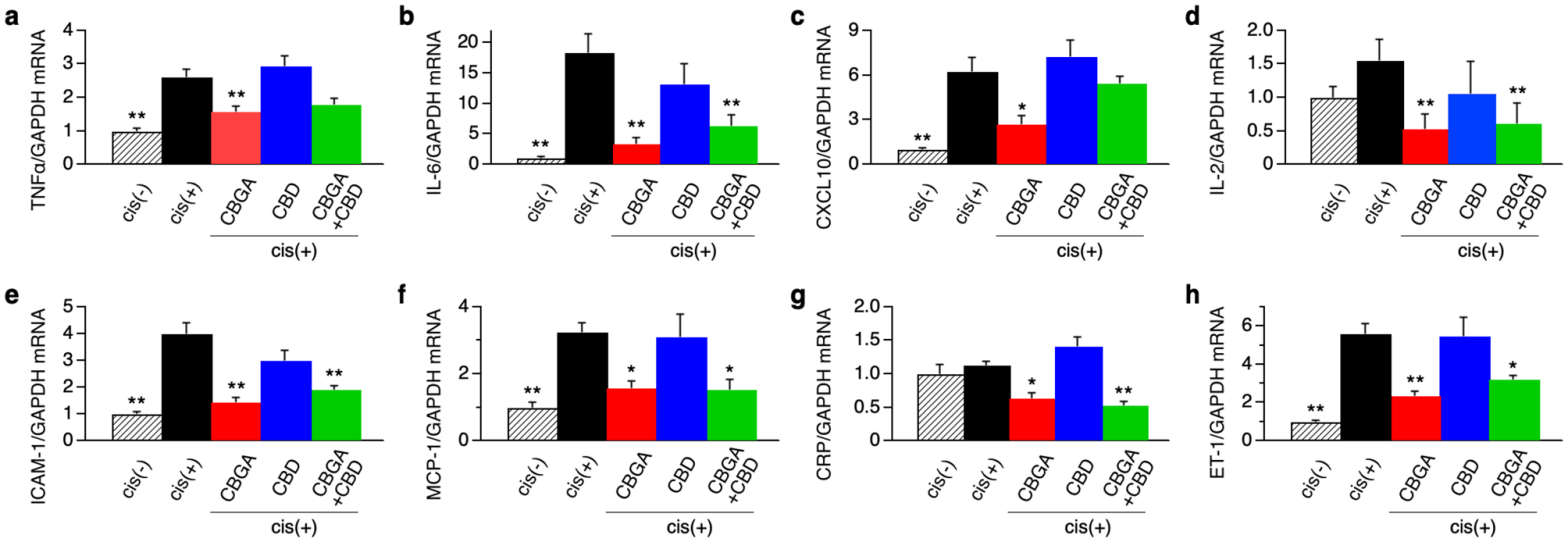
CBGA reduced the mRNA expression of inflammatory cytokines and proteins in cisplatin-induced acute nephropathy. The mRNA levels of TNFα (**a**), IL-6 (**b**), CXCL10 (**c**), IL-2 (**d**), ICAM-1 (**e**), MCP-1 (**f**), CRP (**g**) and ET-1 (**h**) were measured in kidneys of the cisplatin (+) group (black bars), the CBGA treatment group (red bars), the CBD treatment group (blue bars), and the CBGA + CBD treatment group (green bars) at day 3 (see numeric data in Supplementary Table S1). Shaded bars represent the non-cisplatin treatment group as a control group. *p < 0.05, **p < 0.01 vs. cisplatin (+) group.

We next assessed the mRNA levels of inflammatory indicators to determine whether CBGA and CBD attenuate inflammation in cisplatin-induced acute kidney injury. We quantitatively assessed mRNA levels of TNF-α (tumor necrosis factor alpha), IL-6 (interleukin 6), CXCL10 (C-X-C motif chemokine ligand 10), IL-2 (interleukin 2), ICAM-1 (intercellular adhesion molecule 1), MCP-1 (monocyte chemoattractant protein-1), CRP (C-reactive protein), ET-1 (endothelin 1) by normalizing to GAPDH levels. We found that CBGA treatment significantly suppressed mRNA levels of all of these inflammatory cytokines (Fig. 2a–d; red bars) and proteins (Fig. 2e–h; red bars). CBGA suppressed the cisplatin-induced elevated levels of individual mRNA markers by: TNF-α 39.5%, IL-6 82%, CXCL10 57.3%, IL-2 65.8%, ICAM-1 64.2%, MCP-1 51.2%, CRP 43.4%, and ET-1 58.1%. In most cases this reduction returned close to or in some cases below control levels (Fig. 2; red bars and Supplementary Table S1). CBD was generally ineffective in reducing inflammatory markers with the possible exception of mRNA of IL-6, IL-2 and ICAM-1, even though this reduction did not reach statistical significance (Fig. 2b,d,e; blue bars). The combination of CBGA + CBD treatment reduced most mRNA levels similarly to CBGA treatment, but the suppressive effect was somewhat less in some cytokines (Fig. 2; green bars), possibly indicating negative cooperativity effects of these cannabinoids when combined. Together these data demonstrate that CBGA has a stronger inhibitory effect on cisplatin-induced inflammation and kidney damage than CBD.

### CBGA and CBD reduce apoptosis in acute kidney injury

**Figure 3:**
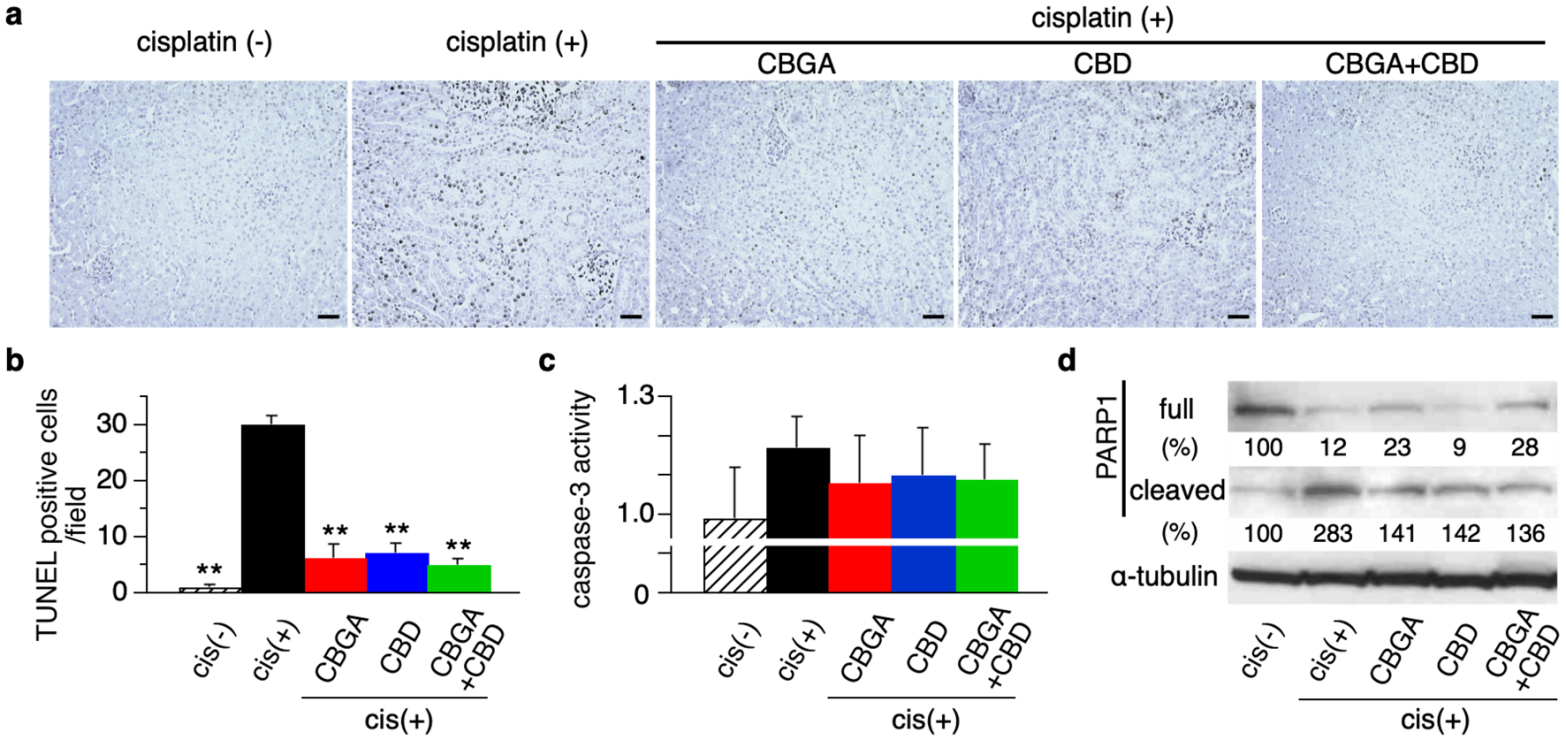
CBGA and CBD reduced apoptosis in cisplatin-induced acute nephropathy. (**a**) Representative pictures of TUNEL-positive apoptotic cells in kidneys from non-cisplatin administered mice or cisplatin-induced nephropathy mice treated with CBGA, CBD or CBGA + CBD (magnification × 200). Scale bars represent 50 µm. (The pictures of high magnification are shown in Supplementary Fig. S1). (**b**) Quantitative analysis of TUNEL-positive renal tubular epithelial cells assessed in kidneys from cisplatin (+) group (black bar), CBGA treatment group (red bar), CBD treatment group (blue bar), CBGA + CBD treatment group (green bar) at day 3. Shaded bar represents the non-cisplatin treatment group as control. (**c**) Caspase-3 activity in kidneys was determined using Caspase-3 assay kit (see “Materials and methods”). (**d**) The levels of full-length PARP1 and cleaved PARP1 protein expression were assessed in kidney tissue from cisplatin-induced acute nephropathy using western blotting (full image is shown in Supplementary Fig. S2). The quantitative percentages are indicated under each picture. **p < 0.01 vs. cisplatin (+) treatment group.

We performed Terminal deoxynucleotidyl transferase dUTP nick end labeling (TUNEL) assays using kidney tissue sections to evaluate the protective effect of CBGA or CBD against apoptosis (Fig. 3a and Supplementary Fig. S1). The number of TUNEL-positive apoptotic cells was increased on tubular epithelial cells in cisplatin-induced nephropathy (Fig. 3b; black bar), but was significantly reduced in mice treated with CBGA, CBD and CBGA + CBD (Fig. 3a,b). We next assessed caspase-3 activity, which also correlates with apoptosis. We observed that cisplatin administration increased caspase-3 activity in kidneys (Fig. 3c; black bar) and this effect was reduced by treatment with CBGA, CBD and CBGA + CBD (Fig. 3c). As a further indicator of apoptotic activity, we assessed the protein levels of poly ADP-ribose polymerase 1 (PARP1), which is an apoptotic marker that is cleaved by caspase-3^31^. The full-length PARP1 was visibly reduced and the cleaved PARP1 increased in cisplatin-induced nephropathy (cisplatin (+) in Fig. 3d). CBGA and CBGA + CBD treatment suppressed this increase of cleaved PARP1 protein. Interestingly, CBD treatment inhibited cleaved PARP1 levels as well as CBGA and CBGA + CBD treatment, however, the full length PARP1 decreased to similar levels as animals treated with cisplatin (+) mice (Fig. 3d). Based on these data, we conclude that CBGA and CBD attenuate cisplatin-induced acute kidney damage by inhibiting apoptosis through blocking of caspase-3 activity and subsequent cleavage of PARP1.

### CBGA and CBD attenuate kidney atrophy in the UUO mouse model

**Figure 4:**
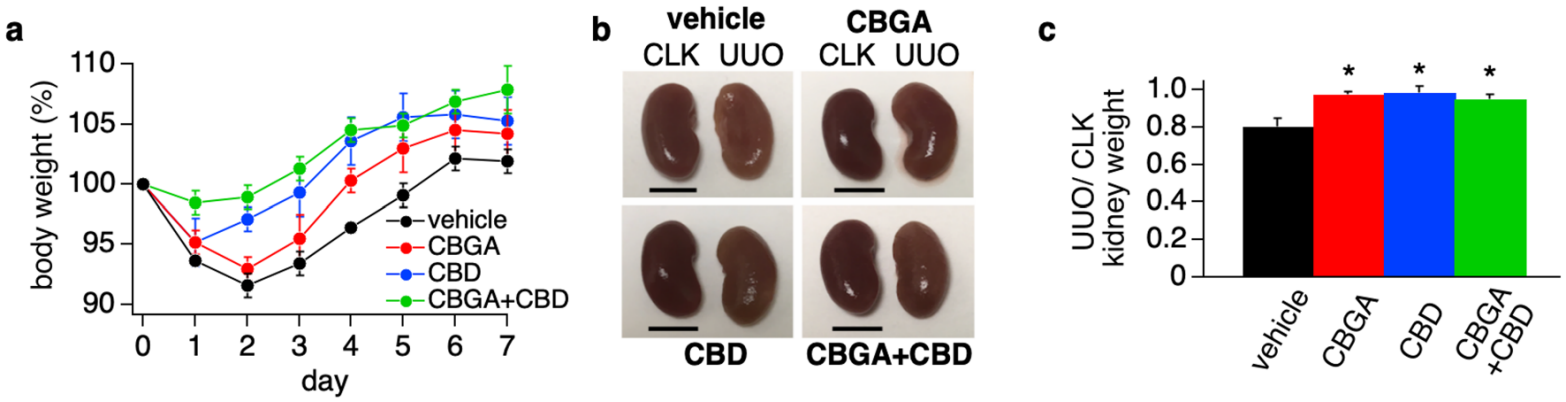
CBGA and CBD prevent kidney atrophy in the UUO mouse model. (**a**) Body weight of mice was measured daily before administration of CBGA (10 mg/kg, red circles), CBD (10 mg/kg, blue circles) and CBGA + CBD (each 10 mg/kg, green circles) from post-UUO surgery day 0 to day 6 (n = 5) and before sacrifice at day 7. The non-treatment group was injected daily with vehicle as a control (n = 4, black circles). Body weight of each day was normalized by the weight before UUO surgery, and the average is indicated. (**b**) Representative images of CLK (left in each picture) and UUO kidneys (right in each picture) isolated from UUO mice at day 7 after UUO surgery. Scale bars represent 5 mm. (**c**) The ratio of UUO kidney weight at day 7. Each UUO kidney weight was normalized by the weight of the corresponding CLK kidney, and the ratio is indicated. Black bar represents the vehicle treatment as a control group, red bar is CBGA treatment group, blue bar is CBD treatment group, and green bar represents CBGA + CBD treatment group. *p < 0.05 vs. vehicle treatment.

We found that CBGA and CBD largely prevent kidney damage in an acute kidney disease model where nephropathy is induced by cisplatin. We next considered whether CBGA and CBD can protect kidneys from chronic nephropathy and progression of renal fibrosis. Since the endocannabinoid system may be involved in renal fibrosis^26,27^ and the cannabinoid receptor 1 (CB1) has been proposed as a therapeutic target against fibrosis^28^, we assessed the effect of CBGA and CBD on renal fibrosis in chronic kidney damage using UUO mice (see “Materials and methods”). We measured body weight daily before cannabinoid administration. UUO mice lost body weight up to day 2 and then fully recovered at day 6 in the vehicle treatment group (Fig. 4a; black circles and Table 2). The CBGA treatment group also lost body weight for 2 days after UUO operation, however, the recovery time was shorter than vehicle treatment group and they were back to their initial body weight before surgery at day 4 (Fig. 4a; red circles). Meanwhile, mice treated with CBD started to recover their body weight at day 2 (Fig. 4a; blue circles) and the combination of CBGA + CBD treatment group prevented the body weight loss entirely (Fig. 4a; green circles).

**Table 2:** Physiological parameters of UUO mice at day 7.

	Body weight (g)	Kidney weight (mg)		Water intake (ml)	Urine (ml)
		CLK	UUO		
Vehicle	20.56 ± 0.20	171 ± 0.4	137 ± 0.8^#^	4.3 ± 0.2	1.24 ± 0.16
CBGA	21.66 ± 0.60	167 ± 0.7	163 ± 0.9	4.7 ± 0.2	1.88 ± 0.17
CBD	22.08 ± 0.45	175 ± 0.7	172 ± 0.8	4.9 ± 0.4	2.01 ± 0.44
CBGA + CBD	22.86 ± 0.21**	175 ± 0.3	167 ± 0.5	4.5 ± 0.4	1.54 ± 0.22

Mice were sacrificed at day 7 after surgery and collected CLK and UUO kidneys (n = 4–5). **p < 0.01 vs. vehicle. ^#^p < 0.05 vs correspondent CLK kidney.

The collected UUO kidneys were flatter and exhibited kidney atrophy with vehicle treatment at day 7 (Fig. 4b, right in each panel). However, treatment of mice with CBGA, CBD and CBGA + CBD combination prevented renal atrophy and maintained their morphology despite UUO (Fig. 4b). In addition, UUO kidneys lost 19.4% weight compared to the corresponding contralateral kidney (CLK) treated with vehicle at day 7 (black bar in Fig. 4c and Table 2), whereas treatments with CBGA, CBD and CBGA + CBD largely prevented the weight loss of UUO kidneys (2.5% in CBGA, 1.4% in CBD, and 4.7% in CBGA + CBD in Fig. 4c).

We also collected urine for 24 h from each mouse and found that the output volume of urine was lower in mice treated with vehicle compared to mice treated with cannabinoids (Table 2), This indicates that kidney function was reduced in UUO mice, but mostly preserved in mice treated with CBGA and CBD. Together, these data suggest that CBGA and CBD treatment have a reno-protective effect in progressive kidney damage and result in less kidney damage and faster recovery from UUO surgery.

### CBGA and CBD reduce the morphologic changes in UUO kidneys

**Figure 5:**
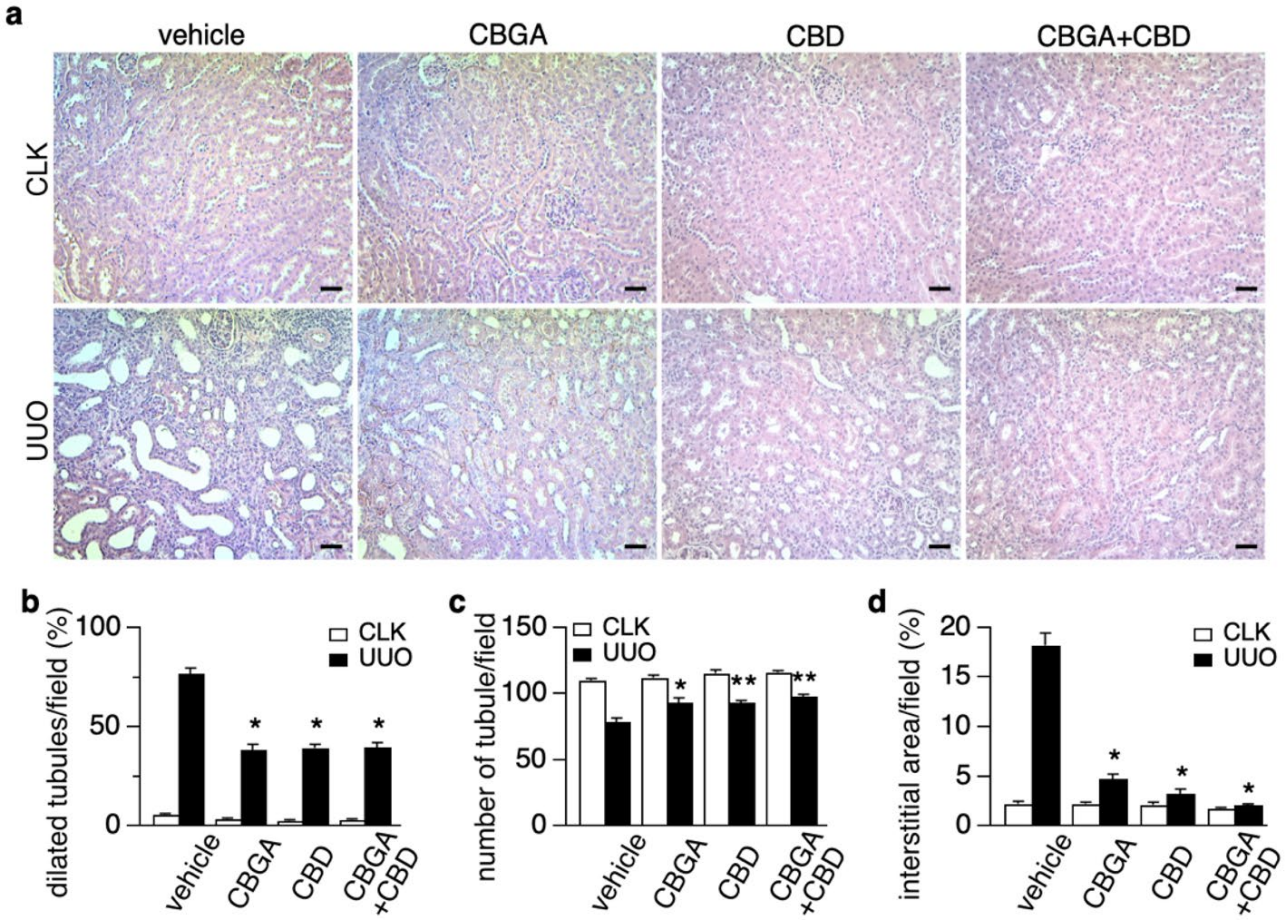
CBGA and CBD protect renal morphology in the UUO kidneys. (**a**) Representative images of HE stainings taken from CLK (upper panels) and UUO (lower panels) kidney sections (magnification × 200) from mice treated with vehicle, CBGA, CBD, CBGA + CBD. Scale bars represent 50 µm. (**b**, **c**) The number of dilated tubules (**b**) and total tubules (**c**) were counted and evaluated statistically in the CLK (white bars) and UUO kidneys (black bars) at day 7 after UUO. (**d**) The percentage of the interstitial area in the CLK (white bars) and UUO kidneys (black bars) were quantified using the sections stained for collagen type I (see Fig. 6a). *p < 0.05, **p < 0.01 vs. UUO kidneys treated with vehicle group.

We next assessed kidney morphology in UUO and CLK kidneys (Fig. 5). We observed that 77.2% of renal tubules dilated in UUO kidneys treated with vehicle, whereas cannabinoid treatment reduced tubular dilation in UUO kidneys by about half compared to vehicle treatment (CBGA: 38.6%, CBD: 39.5%, and CBGA + CBD: 39.7%; Fig. 5a,b; black bars). Since tubular dilation leads to tubule loss in UUO kidneys, we counted the number of tubules to evaluate tubule loss. UUO kidneys lost 28.6% of tubules compared to CLK kidneys in mice treated with vehicle (78.7 in UUO kidneys vs. 110.3 in CLK kidneys; Fig. 5c) and cannabinoid treatment significantly prevented renal tubule loss (Fig. 5c; black bars). Tubule loss was 16.5% in UUO kidney with CBGA treatment (93.4 vs. 111.9 in CLK kidneys; Fig. 5c), 18.6% with CBD treatment (93.9 vs. 115.3 in CLK kidneys; Fig. 5c) and 15.7% with CBGA + CBD treatment (98.1 vs. 116.4 in CLK kidneys; Fig. 5c). We also investigated changes in the interstitium and found that the renal interstitial area increased due to tubule loss in UUO kidneys treated with vehicle (18.2% vs. 2.2% in CLK kidneys; Fig. 5d). Cannabinoid treatment significantly reduced the increase of interstitial area (CBGA: 4.8%, CBD: 3.3%, and CBGA + CBD: 2.1%; black bars in Fig. 5d). Based on these findings, we conclude that CBGA and CBD protect from tubular damage and largely preserve renal morphology in UUO kidneys.

### CBGA and CBD attenuate renal fibrosis in UUO kidneys

**Figure 6:**
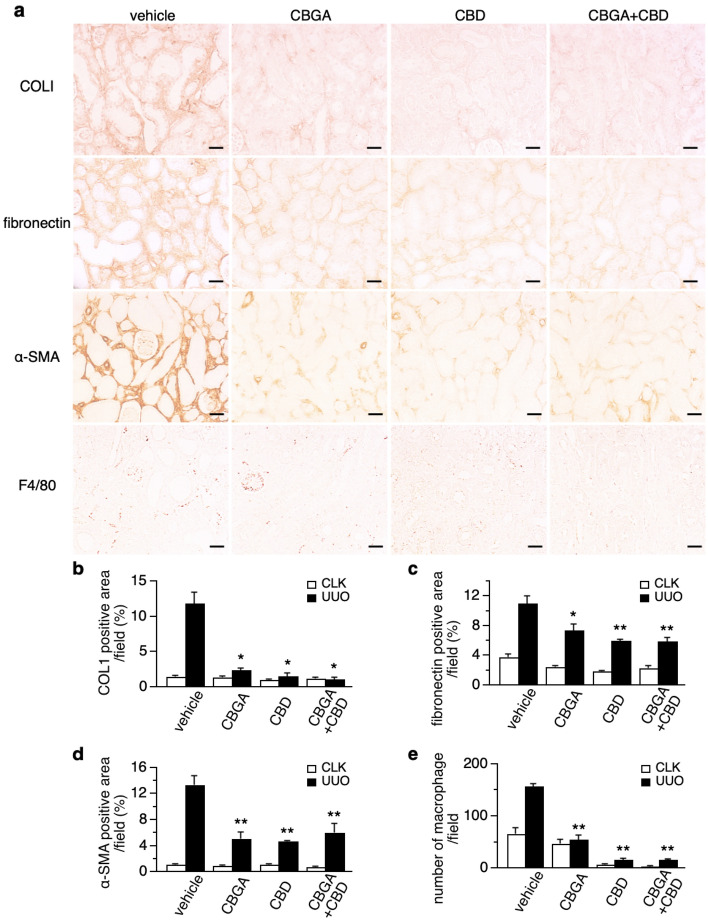
Renal fibrosis is attenuated by CBGA and CBD treatment. (**a**) Representative images of immunostainings for collagen type I, fibronectin, α-SMA and F4/80 from UUO kidneys treated with vehicle, CBGA, CBD and CBGA + CBD (magnification × 200). Scale bars represent 50 µm. (see also Supplementary Fig. S3 for CLK kidneys) (**b–d**) The average percentage of the collagen type I-positive area (**b**), fibronectin-positive area (**c**), α-SMA-positive area (**d**) in CLK (white bars) and UUO kidneys (black bars) with vehicle treatment, CBGA, CBD and CBGA + CBD treatment. The intensity of staining in the interstitium was computed using ImageJ software in 10 representative non-overlapping slides. (**e**) F4/80 antibody was used to detect macrophage on immunostaining, F4/80-positive cells in interstitium were counted as number of macrophages. *p < 0.05, **p < 0.01 vs. UUO kidneys treated with vehicle group.

Since CBGA and CBD had a protective effect on tubular damage in Fig. 5, we evaluated whether CBGA and CBD could suppress renal fibrosis in UUO kidneys. We performed immunohistochemistry with antibodies of collagen type I, fibronectin and α-smooth muscle actin (α-SMA) as markers of renal fibrosis and quantified the intensity of immunostaining. Collagen type I-positive staining increased by 11.9% in UUO kidneys treated with vehicle (Fig. 6a,b; black bar), and this was suppressed with treatments of CBGA to 2.4%, CBD to 1.6% and the combination of CBGA + CBD treatment to just 1.2% vs 1.1% in CLK kidneys (Supplementary Fig. S3 and Fig. 6b, white bar). Fibronectin also increased by 11% in UUO kidneys treated with vehicle (Fig. 6a,c; black bar), and CBGA, CBD or combination treatments decreased UUO-mediated fibronectin levels to 7%, 6% and 5.9%, respectively (Fig. 6a,c; black bars). Additionally, the increase in α-SMA seen in vehicle-treated UUO kidneys was similarly reduced by cannabinoid treatments (Fig. 6a,d; black bars). Representative images of these fibrotic markers in CLK kidneys are shown in Supplementary Fig. S3, illustrating that positive areas exhibited very low levels, with no significant difference between each group of treatment (Fig. 6b–d; white bars). Complementing the histochemical data, we assessed α-SMA protein levels by western blotting of kidney tissue samples, where we confirmed that α-SMA strongly increased in UUO kidneys with vehicle treatment but was significantly suppressed by treatment of CBGA, CBD or CBGA + CBD (Supplementary Fig. S4).

Since macrophages infiltrate inflamed and damaged kidneys, we performed immunostaining using F4/80 antibody and assessed the number of macrophages. Macrophage numbers significantly increased in UUO kidneys with vehicle treatment compared to CLK kidneys (158 vs. 66; Fig. 6a,e and Supplementary Fig. S3), whereas cannabinoid treatment significantly reduced invasive macrophage numbers in UUO kidneys (55 in CBGA, 17 in CBD, 16 in CBGA + CBD; Fig. 6e; black bars). CBD and CBD + CBGA also reduced macrophage numbers in CLK kidneys (6.7 and 4 macrophages, respectively; Fig. 6e, white bars). Taken together, we conclude that CBGA and CBD inhibit the build-up of extracellular matrix and reduce the amount of renal fibrosis as well as macrophage infiltration following UUO kidney damage.

### CBGA inhibits TRPM7 membrane currents and protein expression in cisplatin-induced nephropathy

**Figure 7:**
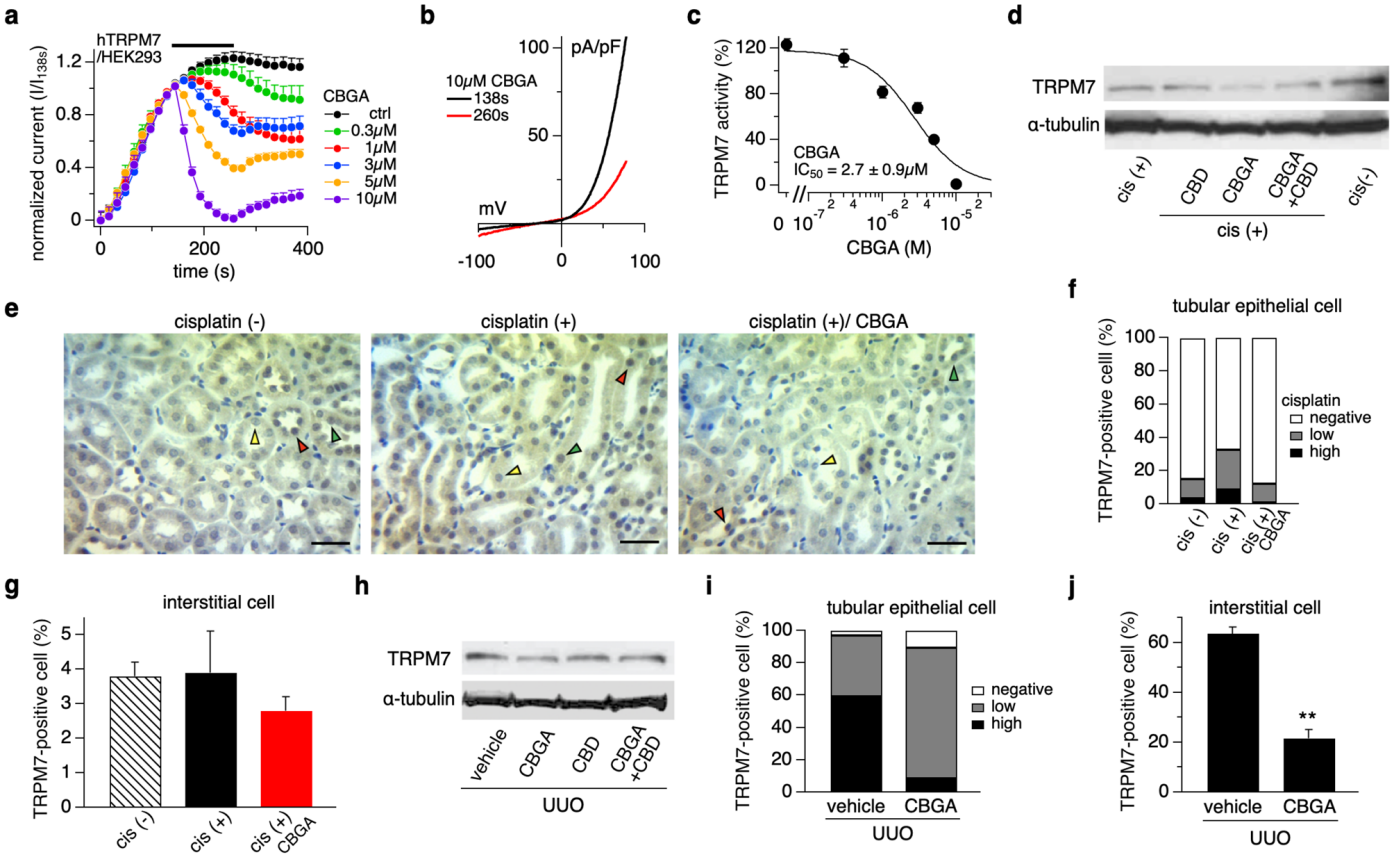
CBGA inhibits TRPM7 currents in HEK293 cells and reduces TRPM7 expression in kidney damage. (**a**) Inhibitory effects of CBGA on TRPM7 currents was investigated by whole-cell patch-clamp recordings from HEK293 cells overexpressing TRPM7. Average TRPM7-mediated outward currents at + 80 mV extracted from ramp currents delivered at 0.5 Hz and plotted as a function of time. Concentrations of 0.3 µM (green circles, n = 5), 1 µM (red circles, n = 7), 3 µM (blue circles, n = 7), 5 µM (orange circles, n = 7), and 10 µM (purple circles, n = 6), were applied from 140 to 260 s (black bar). Standard Ringer without CBGA contained acetonitrile as vehicle and was used as control (black circles, n = 8). (**b**) Average I/V curve of TRPM7 currents extracted before (138 s, black line) and after (260 s, red line) 10 µM CBGA application. (**c**) The dose–response curve of CBGA on TRPM7 channels. Data points for the curve were obtained from the normalized currents in panel (**a**) at 260 s. (**d**) TRPM7 protein expression was examined in kidney tissues from cisplatin-administered mice treated with CBD, CBGA and CBGA + CBD using western blotting (Full images are shown in Supplementary Fig. S5a). (**e**) Representative pictures of immunostaining to TRPM7 (magnification × 400) in kidneys from non-cisplatin treated mice (left panel), cisplatin-treated mice (middle panel) and cisplatin/CBGA-treated mice (right panel). Scale bars represent 50 µm. Green arrow heads indicate TRPM7-low expression and red arrow heads are high expression levels of TRPM7, yellow arrow heads indicate TRPM7-negative (TRPM7 staining not detectable) in renal tubular epithelial cells. (**f**) The percentage of TRPM7-positive cells in tubular epithelial cells in kidneys from cisplatin-treated mice. TRPM7-high expression (black bars), TRPM7-low expression (gray bars) and non-detectable TRPM7 staining (white bars) in tubular epithelial cells assessed in a total of 10 non-overlapping fields. (**g**) The percentage of TRPM7-positive interstitial cells assessed in the same fields as in panel (**f**). (**h**) TRPM7 protein expression was examined in cortical UUO kidney tissue from mice treated with vehicle, CBGA, CBD and CBGA + CBD using western blot (Full images are shown in Supplementary Fig. S6a). (**i**) The percentage of TRPM7 in tubular epithelial cells from UUO kidneys treated with CBGA or not. Same experimental protocol as in (**f**). Representative pictures are shown in Supplementary Fig. S6c. (**j**) The percentage of TRPM7-positive interstitial cells assessed in the same fields as in panel (**i**). **p < 0.01 vs. vehicle treatment group.

We have recently reported that CBGA suppresses IL-2 production in T cells by inhibiting Store-Operated Calcium Entry (SOCE)^23^ and previously found that Transient Receptor Potential (TRP) Melastatin 7 (TRPM7) regulates SOCE^32^. In the context of kidney disease, TRPM7 expression increases in UUO kidneys and systemic application of NS8593, a known TRPM7 inhibitor, prevents kidney atrophy in UUO kidneys, retains tubular formation, and reduces TRPM7 expression to normal levels^33^. Therefore, we considered the possibility that CBGA might inhibit TRPM7 channels. We assessed the inhibitory efficacy of CBGA on TRPM7 currents using patch-clamp recordings in human TRPM7 stably and inducibly overexpressed in human embryonic kidney HEK293 cells and found that CBGA indeed blocked TRPM7 channels in a dose-dependent manner (Fig. 7a,b) with a half-maximal inhibitory concentration (IC_50_) of 2.7 ± 0.9 µM (Fig. 7c).

In cisplatin-treated mice, cisplatin reduced overall kidney TRPM7 protein expression (Fig. 7d). CBGA and CBGA + CBD treatment further reduced TRPM7 expression, although CBD was less effective in doing so (Fig. 7d). Interestingly, TRPM7 mRNA was up-regulated by CBGA but not by CBD (Supplementary Fig. S5b), possibly in an attempt to compensate for the loss of functional TRPM7 protein. We also performed immunohistochemistry using anti-TRPM7 antibodies and assessed TRPM7 protein expression on individual renal tubular epithelial cells and interstitial cells in cisplatin-induced kidney injury (Fig. 7e) with or without CBGA treatment. We distinguished 3 patterns of TRPM7 staining in tubular epithelial cells: a strong positive staining (TRPM7-high expression, red arrow heads in Fig. 7e), a slight positive staining (TRPM7-low expression, green arrow heads in Fig. 7e), and TRPM7 negative staining (TRPM7-no expression, yellow arrow heads in Fig. 7e, cell appearing blue).

Quantification of these data in cisplatin-untreated mice showed that 11.6% of epithelial cells had TRPM7-low expression (Fig. 7f, gray bar), 3.4% of cells showed highly expressed TRPM7 (Fig. 7f, black bar) and the remaining 84.9% cells had undetectable TRPM7 staining (Fig. 7f, white bar). In cisplatin-treated mice, TRPM7-positive cells increased in tubular epithelial cells, where 24.1% displayed TRPM7-low expression and 8.7% had highly expressed TRPM7 (Fig. 7f). CBGA treatment of cisplatin-exposed mice reduced TRPM7-high positive tubular epithelial cells to just 1.1% and reduced TRPM7-low cells to 11.3% (Fig. 7f). Similarly, as illustrated in the supplemental data, cisplatin up-regulated TRPM7 mRNA in a cultured epithelial cell line from normal rat kidney (NRK-52E) but not in fibroblasts (NRK-49F, Supplementary Fig. S5c). Conversely, the percentage of TRPM7-expressing interstitial cells was very low and here there was no significant difference between cisplatin-untreated (3.8%; Fig. 7g, shaded bar), cisplatin-exposed mice (3.9%; Fig. 7g, black bar), and mice treated with cisplatin plus CBGA (2.8%; Fig. 7g, red bar).

We also assessed the level of TRPM7 protein expression in the UUO model. Here, TRPM7 protein was slightly reduced in UUO kidneys treated with CBGA (Fig. 7h), although there was no significant difference in TRPM7 mRNA levels between vehicle or CBGA treatment (Supplementary Fig. S6b). We also performed TRPM7 immunostaining in UUO kidneys (Supplementary Fig. S6c) and found that 59.6% of tubular epithelial cells had high TRPM7 expression (Fig. 7i, black bar), 37.6% displayed TRPM7-low expression (Fig. 7i, gray bar), and 2.8% had no detectable TRPM7 expression (Fig. 7i, white bar). CBGA treatment decreased TRPM7-high expressing cells to 8.6%, while TRPM7-low expressing cells increased to 81.0%. The total numbers of TRPM7-positive cells of tubular epithelial cells (TRPM7-high and TRPM7-low) was 89.6% (vs. 97.2% in vehicle control, Fig. 7i). Thus, it appears that CBGA did not significantly reduce the number of TRPM7-positive cells, but instead reduced TRPM7 expression overall and shifted the proportion of highly expressing cells into cells with lower TRPM7 expression. In contrast to the cisplatin model, UUO kidneys massively increased TRPM7-positive interstitial cells to 63.7%, and this effect was significantly decreased to 21.7% in UUO kidneys treated with CBGA (Fig. 7j). Taken together, these results suggest that the two experimental models (acute cisplatin vs chronic UUO challenge) differ in that TRPM7 expression is differentially regulated in tubular and interstitial cells. Moreover, it appears that the reno-protective effects of CBGA might be at least partially through reduced TRPM7 expression, but apparently targeting different cell types in the two kidney disease models. Thus, CBGA reduced TRPM7 in tubular epithelial cells in cisplatin-induced acute inflammatory kidney injury at day 3, whereas it reduced TRPM7 in interstitial cells in UUO kidneys undergoing renal fibrosis at day 7.

## Discussion

In this study, we found that the cannabinoids CBGA and CBD reduced or prevented kidney damage in both acute and chronic nephropathic mouse models induced by cisplatin and unilateral ureteral obstruction, respectively. However, they did so in a differential and specific manner, likely due to differential mechanisms of action.

Cisplatin induces renal inflammation, tubular cell damage, and loss of kidney homeostasis and function, resulting in the reduction of urine output volume. Both CBGA and CBD prevented the reduction of output urine, indicating that kidney function was maintained by either cannabinoid. Consistent with this result, both CBGA and CBD as well as their combination also suppressed the increases in BUN and creatinine seen in cisplatin-treated mice. Cisplatin is used as a chemotherapeutic in cancer treatments and two of its major side effects include hypomagnesemia and kidney damage^34,35^. Thus, magnesium supplementation is recommended to prevent kidney injury before cisplatin chemotherapy. The magnesium-permeable ion channel TRPM7 plays a key role in cellular and organismal magnesium homeostasis and the TRPM7 inhibitor waixenicin A decreases intestinal magnesium absorption and leads hypomagnesemia in vivo^36^. Moreover, TRPM6, a homologous channel that can form heteromeric TRPM6/TRPM7 channel assemblies, serves magnesium regulation in kidney^37^. Accordingly, hypomagnesemia occurs in kidney-specific TRPM6-deficient mice due to renal magnesium wasting^38^. Therefore, we considered that CBGA might induce hypomagnesemia in vivo through TRPM7 blockage. However, mice did not have exhibit hypomagnesemia at day 3 after we injected CBGA 3 times, even though CBGA suppressed TRPM7 expression in nephropathic kidneys in this study (Supplementary Fig. S7). Possible reasons for the lack of hypomagnesemia include potential compensation by the sister channel kinase TRPM6 or other magnesium transporters. In addition, it is possible that the CBGA concentrations reached in vivo may not have sufficed to reduce TRPM7 magnesium transport to cause hypomagnesemia either through channel block or protein downregulation or both.

CBGA treatment also prevented body weight loss and general kidney inflammation, significantly reducing cisplatin-induced increases in numerous inflammatory cytokines and protein markers (Fig. 2). Interestingly, although CBD has been reported to possess anti-inflammatory effects, we did not observe a strong inhibitory effect on cisplatin-induced acute kidney inflammation. Since CBGA potently blocks TRPM7 and has also been shown to inhibit store-operated calcium entry (SOCE) in various pro-inflammatory immune cells^23^, this combination of effects may contribute significantly to the kidney-protective effect of CBGA observed in the cisplatin nephropathy model. While CBD was also protective in this nephropathy model, its effects on TRPM7 (Supplementary Fig. S8) and SOCE^23^ were far less potent, indicating that alternative, yet to be determined mechanisms might underlie its protective effects.

CBGA also reduced renal fibrosis in the UUO renal fibrotic model, but the reno-protective effect of CBGA seems less than that of CBD treatment. This is evident from the speed of recovery after surgery, where CBGA treatment led to faster recovery from body weight loss after UUO surgery compared to the mouse group with vehicle treatment, but CBD was even more protective. The differences observed between the two cannabinoids in the two nephropathy models are likely due to both differences in pathological mechanisms engaged by the experimental models as well as differences in pharmacological targets of CBD and CBGA. It is also possible that these differences might be partially explained by pharmacokinetics as both molecules might have different bioavailabilities, and CBGA has been shown to strongly bind to serum albumin^23^.

Cisplatin-induced nephropathy represents a model of acute inflammatory kidney injury. Cisplatin treatment results primarily in tubular epithelial cell damage, where TRPM7 is up-regulated in tubular epithelial cells, but not in interstitial cells. Because TRPM7 can lead to cell death when its activity is up-regulated and cells experience calcium overload^39^, it is conceivable that CBGA may protect tubular epithelial cells by preventing TRPM7 up-regulation in these cells. In contrast to the cisplatin model, UUO represents a chronic kidney disease model with progressive renal fibrosis that has not only tubular epithelial cell damage but also results in a massive increase in the number of interstitial cells due to kidney damage. However, tubular epithelial cells still significantly outnumber the interstitial cell population at day 7 in UUO kidneys (Supplementary Fig. S6d). Thus, in UUO kidneys, kidney injury increases tubular epithelial cell proliferation and induces epithelial-to-mesenchymal transition (EMT), where interstitial cells such as fibroblasts and myofibroblasts increase in interstitium by EMT and proliferation. These myofibroblasts produce extracellular matrix and contribute to progressive renal fibrosis. Therefore, the protection of tubular epithelial cells is important to prevent EMT and the increase of interstitial cell number for reno-protection.

We previously reported that NS8593, a known TRPM7 inhibitor^40^, blocks TRPM7 in tubular epithelial cells in UUO kidneys and reduces renal fibrosis^33^. In the present study, we found that CBGA suppresses TRPM7 expression in both interstitial cells and tubular epithelial cells in UUO kidneys. However, while CBGA merely lowered TRPM7 levels in tubular epithelial cells by shifting the cell population with TRPM7-high expression levels into the TRPM7-low expression population rather than increasing entirely TRPM7-negative cells. Thus, the overall TRPM7 protein did not decrease strongly in the western blot that reflects the whole kidney. On the other hand, in cisplatin-induced nephropathy, CBGA mainly inhibited TRPM7 expression in tubular epithelial cells, and western blots therefore revealed a decrease in total TRPM7 protein in cisplatin-exposed mice treated with CBGA.

When comparing the two kidney nephropathy models, we propose that CBGA-induced inhibition of TRPM7 may progressively shift its impact on different target cell populations depending on the stage of progressive kidney damage: In the acute stage of nephropathy, the damage will mostly involve tubular epithelial cells, whereas the late stage will mainly involve progressive renal fibrosis. In the present study, we used a CBGA dose of 10 mg/kg, at which it obviously had a reno-protective effect on acute kidney injury induced by cisplatin. However, it might need a higher dosage of CBGA to protect kidney from chronic kidney disease with progressing renal fibrosis. Conversely, while CBD had an anti-fibrotic effect in UUO kidneys and preserved kidney function in cisplatin-induced kidney at 10 mg/kg of CBD, this dose may not be enough to suppress acute inflammation in kidney. The reason for this could be that CBD is not nearly as potent as CBGA in suppressing SOCE in pro-inflammatory immune cells^23^.

In summary, CBGA has protective and anti-inflammatory effects in kidney damage likely through its inhibitory on TRPM7 and SOCE in the early stages of nephropathy and also suppresses renal fibrosis seen in chronic kidney disease model. CBD also suppressed renal fibrosis, although it does not appear to have a strong effect of anti-inflammation in acute nephropathy, consistent with its rather poor efficacy in blocking TRPM7 or SOCE. CBGA has the potential to protect against kidney damage from inflammation and fibrosis in both acute and chronic kidney disease, while CBD is partially protective against the progression of chronic renal fibrosis. CBGA and CBD alone or in combination could be helpful as therapeutic options to treat chronic kidney disease with anti-inflammatory and anti-fibrotic properties and CBGA may be able to serve as an adjuvant for cisplatin chemotherapy.

## Materials and methods

### Animals and ethics statement

Male C57Bl/6 mice 9 weeks of age, weighing 21 to 27 g at the start of the experiment were used for cisplatin-induced nephropathy model. For the UUO model, C57Bl/6 mice 6 weeks of age and weighing 18 to 23 g at the start of the experiment were used. Mice were purchased from Charles River Laboratories and housed at an ambient temperature of 20 ± 1 °C and a 12-h light/dark cycle, allowing free access to food and water. The experimental procedures were reviewed and approved by the Institutional Animal Care and Use Committee of the University of Hawaii (#17-2527-4) and the Animal Care Committee at The Queen’s Medical Center (#RA-2017-008). All procedures were in accordance with guidelines recommended by the NIH and reported in accordance with ARRIVE guidelines.

### Reagents

CBGA and CBD were purchased from Cayman Chemical Company and dissolved in EtOH. They were prepared 2 mg/ml in a vehicle of ethanol/Tween80 (G Biosciences)/saline (1:1:18) before injection. Cisplatin was purchased from Sigma and kept as a stock solution of 1 mg/ml dissolved in saline.

### Cisplatin-induced nephropathy model

Cisplatin was administered to mice (16 mg/kg) by intraperitoneal (i.p.) injection. Comparing cisplatin-induced kidney damage to non-cisplatin administered kidney, an equal volume of vehicle saline solution (16 ml/kg) was administered by i.p. injection as a negative control (cisplatin (−), n = 4). CBGA or CBD (10 mg/kg) or combination of CBGA + CBD (10 mg/kg each) were injected 2 h before cisplatin injection and then administered at 24 and 48 h after cisplatin injection (n = 5). This dose has previously been used in animal models by numerous studies (e.g., Rock et al.^19^). For vehicle controls, an equal volume of vehicle solution (5 ml/kg) was administered by i.p. injection in cisplatin-treated mice (cisplatin (+), n = 5) and cisplatin (−) mice. Mice were transferred to metabolic cages after 48 h of cisplatin administration and urine was collected for 24 h. The output volume of urine was measured, then stored at − 80 °C for kidney function assays. Mice were euthanized by isoflurane inhalation after 72 h of cisplatin administration. Blood and kidneys were collected and subjected to further evaluations.

### UUO mouse model

Unilateral ureteral obstruction (UUO) was created by ligating the left ureter with 3–0 silk through a left lateral incision under anesthesia of isoflurane^7,33,41^. CBGA (10 mg/kg) or CBD (10 mg/kg) or the combination of CBGA + CBD (10 mg/kg each) were administered to UUO mice by i.p. injection daily for 7 days with the first injection starting directly after surgery (n = 5). For vehicle controls, the non-treatment group received an equal volume of vehicle solution (5 ml/kg body weight, ethanol/Tween80/saline 1:1:18) was administered daily by i.p. injection (n = 4). Mice were sacrificed using inhalation of isoflurane at day 7 after surgery. Obstructed left kidneys (UUO) and non-obstructed contralateral right kidneys (CLK) were collected and subjected to the experiments described below.

### Kidney function assay

Blood was collected by cardiac puncture after mice had been euthanized. Using collected urine and serum, creatinine and blood urea nitrogen (BUN) value were measured to evaluate kidney function using commercial kits (BioAssay System).

### Histopathological evaluation

Kidney tissues were cut and fixed in 4% paraformaldehyde in phosphate-buffered saline at 4 °C overnight. Kidneys were then embedded in paraffin and sectioned. Kidney tissue sections (5 µm thickness) were rehydrated and stained with hematoxylin and eosin staining for histopathological analysis. Tubular injuries were semi-quantitatively graded 0 to 4 evaluating injured tubules that showed necrosis and tubular dilation in 10 non-overlapping areas of the outer stripe of the outer medulla as follows; 0, none (0%); 1, weak (≤ 20%); 2, mild (> 20 to ≤ 50%); 3, moderate (> 50 to ≤ 80%); 4, strong (> 80%).

### RNA isolation and qRT-PCR assay

Collected kidneys were treated with RNALater (Qiagen) at 4 °C overnight and then stored at − 80 °C to extract RNA and perform qRT-PCR. Total RNA was extracted from kidney tissues using RNeasy Protect Mini Kit (Qiagen) and reverse transcription of RNA was performed to make cDNA using the SuperScript III First-Stand Synthesis System for RT-PCR kit (Invitrogen) with 1 μg of total RNA. cDNA was subjected to qRT-PCR for amplification and online quantification using QuantStudio 12 K Flex Real-Time PCR System (Applied Biosystems). All PCR experiments used SYBR Green. The primer pairs were purchased from Qiagen and Sigma. GAPDH was used as internal control and the ratio of each target mRNA was normalized by GAPDH mRNA.

### Evaluation of apoptosis

Deoxynucleotidyl transferase-mediated dUTP nick end labeling (TUNEL) was performed to detect apoptotic cell death using an ApopTag Peroxidase In Situ Apoptosis Detection Kit (Chemicon). For each kidney, the number of apoptotic tubular epithelial cells in 20 non-overlapping areas of the outer stripe of the outer medulla were counted under × 400 magnification. The number of TUNEL-positive nuclei was averaged for each mouse.

### Caspase-3 assay

Caspase-3 activity was measured by Caspase-3 colorimetric assay kit (BioVison) following the manufacturer’s instructions. Kidney tissue was homogenized in cell lysis buffer and lysates were centrifuged at 13,000 rpm for 15 min at 4 °C and protein concentrations of lysates were measured using a protein assay reagent (Bio-Rad Laboratories). DTT (10 mM) and DECD-pNA substrate (200 µM) were added to equal amounts of protein sample (200 μg) and then incubated at 37 °C for 1 h. Samples were measured 405 nm absorbance using a microplate reader (Bio-Rad).

### Western blot analysis

Cortical kidney tissues were homogenized in radioimmunoprecipitation assay (RIPA) buffer (25 mM Tris-HCI pH7.4, 150 mM NaCl, 0.1% SDS, 0.5% Triton-X100, 0.5% sodium deoxycholate) containing protease inhibitor cocktail (Sigma). After incubation for 30 min on ice, lysates were centrifuged at 13,000 rpm for 15 min at 4 °C and protein concentrations of supernatant were measured in the same manner as described for the caspase-3 assay^7,41^. Soluble lysates were boiled with Bolt LDS sample buffer (Invitrogen) at 98 °C for 5 min. Equal amounts of proteins (50 μg) were loaded and separated in Bolt 4–12% gel (Invitrogen) then transferred to PVDF membrane (Millipore). Proteins were detected using the antibodies of rabbit polyclonal anti-human PARP (Cell Signaling, 9542), anti-human TRPM7 (MyBioSource, MBS9214600), mouse monoclonal anti-human α-SMA (Dako, M0851), anti-human α-tubulin (Novus Biologicals, NB100-690). α-tubulin was used as control. The antibody-bound proteins were visualized using an enhanced chemiluminescence system (Bio-Rad Laboratories) or Odyssey CLx Imaging System (Li-COR).

### Immunohistochemical analysis

Kidney tissue slices were also used for immunohistochemistry studies to determine the expression of targeted proteins such as collagen type Ι, fibronectin, α-SMA, F4/80 and TRPM7 using a VECTASTAIN ABC kit (Vector laboratories) as a standard biotin–streptavidin–peroxidase method. Antigen retrieval was performed in 10 mM citrate buffer (pH 6.0) using a microwave for 15 min. Endogenous peroxidase activity was blocked in 3% hydrogen peroxide for 10 min. The primary antibodies were as follows: rabbit polyclonal anti-human collagen type Ι (Abcam, ab21286), anti-human fibronectin (Abcam, ab23750), anti-human TRPM7 (MyBioSource), rabbit monoclonal anti-mouse F4/80 (Abcam, ab111101), and mouse monoclonal anti-human α-SMA (Dako). The secondary antibodies were affinity-purified biotinylated goat anti-rabbit or anti-mouse immunoglobulin with ImmPACT DAB kit (Vector laboratories) as peroxidase substrates. For TRPM7 staining, the kidney sections were slightly counterstained with hematoxylin. The collagen type Ι-, fibronectin- and α-SMA-positive interstitial areas were quantified in 10 randomly selected, non-overlapping renal cortical fields at × 200 magnification using ImageJ software. We set a threshold to compute the positive areas for each field and computed the ratio of the positive areas over the whole interstitial area. The interstitial area was measured using collagen type Ι staining slides. F4/80-positive cells were counted in interstitium. The number of TRPM7-positive tubular epithelial and interstitial cells were counted in 10 randomly selected, non-overlapping renal cortical fields at × 200 magnification, and the mean values were obtained. The number of tubules, tubular epithelial cells, and interstitial cells were counted using the kidney sections stained for TRPM7.

### Cell culture

Tetracycline (Tet)-inducible HEK293-TREx cells, stably transfected with human TRPM7, were cultured in DMEM medium (Gibco) containing 10% fetal bovine serum (FBS, Gibco), zeocin (0.4 mg/ml, Invitrogen) and blasticidin (5 µg/ml, Invitrogen). Overexpression was induced adding 1 µg/ml tetracycline (Gibco) to the culture medium. Patch-clamp experiments were performed 18–22 h post-tetracycline induction. The normal rat kidney fibroblast cell line NRK-49F and epithelial cell line NRK-52E (ATCC) were maintained in DMEM medium (ATCC) supplemented with 5% fetal calf serum (ATCC). Cells were maintained at 37 °C under 5% CO_2_ condition.

### Electrophysiology

For whole-cell patch-clamp experiments assessing overexpressed human TRPM7, the extracellular solution contained (in mM): 140 NaCl, 2.8 KCl, 2 MgCl_2_, 1 CaCl_2_, 10 HEPES–NaOH, 11 Glucose (pH 7.2, 300 mOsm). Intracellular pipette-filling solutions contained (in mM): 120 Cs-glutamate, 8 NaCl, 10 Cs-Bapta, 1 Na-ATP, 1.76 MgCl_2_ (860 µM Mg-ATP, 700 µM free Mg^2+^), 10 HEPES-CsOH (pH 7.2, 300 mOsm). All salts were from Sigma, free intracellular Mg^2+^ concentration was calculated with WebMaxC Standard. CBGA was dissolved in acetonitrile as stock solution and stored at − 20 °C, diluted to 0.3, 1, 3, 5, 10 µM with the extracellular solution used for patch-clamp experiments. As a control application, acetonitrile was diluted in the extracellular solution. Patch clamp experiments were performed in the whole-cell configuration using an EPC-9 amplifier (HEKA, Germany), TRPM7 currents were elicited by a ramp protocol from − 100 to + 100 mV from a holding potential of 0 mV over 50 ms and acquired at 0.5 Hz. Outward current amplitudes over the course of the experiment were extracted at + 80 mV and plotted versus time. Data were normalized to the time point before cannabinoid application. All values are given as mean ± standard error of mean (SEM). Patch glass pipettes (Sutter Instrument) were pulled and polished, and had a tip resistance of 2–3 MΩ with the solutions used.

### Statistical analysis

All values were given as means ± SEM. Statistical analysis was performed by Statview (version 4.0). Differences between groups were examined for statistical significance by analysis of variance (ANOVA) then used Bonferroni-Dunn as a post-hoc test. Values of p < 0.05 were considered as statistically significant.

## Supplementary Information

Supplementary Information.

## Data Availability

The data that support the findings of this study are available from the corresponding author upon request.
