# Supplementary material for: CBGA ameliorates inflammation and fibrosis in nephropathy
Source: Sci Rep. 2023 Apr 18;13:6341. doi: 10.1038/s41598-023-33507-2 (PMC10113213; doi:10.1038/s41598-023-33507-2)
Supplement: Supplementary file 1 — Supplementary Information. [file 41598_2023_33507_MOESM1_ESM.pdf]

Supplementary Information

CBGA ameliorates inflammation and fibrosis in nephropathy

Sayuri Suzuki[1*], Andrea Fleig[1,2,3], Reinhold Penner[1,2,3]

[1] Center for Biomedical Research, The Queen's Medical Center, 1301 Punchbowl St., Honolulu, HI 96813, U.S.A.
[2] University of Hawaii Cancer Center, 651 Ilalo St., Honolulu, HI 96813, U.S.A.
[3] John A. Burns School of Medicine, University of Hawaii, 651 Ilalo St., Honolulu, HI 96813, U.S.A.
*e-mail: sayuris@hawaii.edu

Table S1: Quantitative mRNA levels of inflammatory cytokines and proteins in cisplatin-induced acute nephropathy.

			cis (+)		
	cis (-)	cis (+)	CBGA	CBD	CBGA+CBD
TNFα	1 ± 0.09	2.61 ± 0.23	1.58 ± 0.16	2.93 ± 0.30	1.79 ± 0.18
IL-6	1 ± 0.31	18.38 ± 3.17	3.31 ± 1.00	13.18 ± 3.40	6.37 ± 1.77
CXCL10	1 ± 0.09	6.27 ± 0.97	2.68 ± 0.60	7.26 ± 1.10	5.46 ± 0.50
IL-2	1 ± 0.17	1.55 ± 0.32	0.53 ± 0.23	1.06 ± 0.48	0.62 ± 0.30
ICAM-1	1 ± 0.10	3.99 ± 0.40	1.43 ± 0.18	2.99 ± 0.38	1.91 ± 0.16
MCP-1	1 ± 0.17	3.24 ± 0.28	1.58 ± 0.22	3.10 ± 0.68	1.54 ± 0.31
CRP	1 ± 0.14	1.13 ± 0.06	0.64 ± 0.08	1.41 ± 0.14	0.53 ± 0.06
ET-1	1 ± 0.10	5.58 ± 0.55	2.34 ± 0.26	5.49 ± 0.99	3.23 ± 0.21

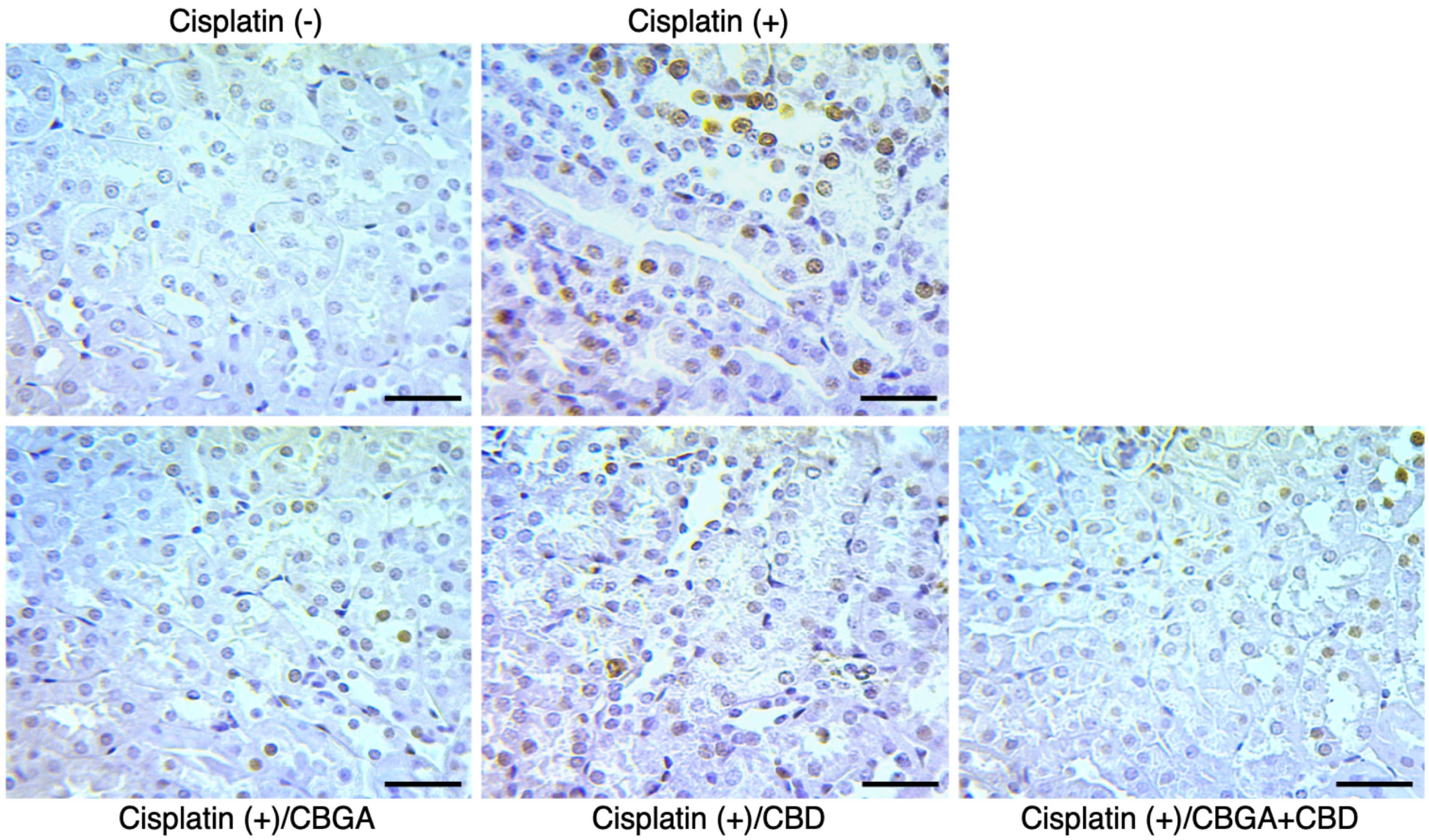

Fig. S1 Representative high-magnification images of TUNEL-positive apoptotic cells in kidneys from cisplatin-induced nephropathy model treated with CBGA, CBD and CBGA+CBD (magnification x400). Scale bars represent 50 µm.

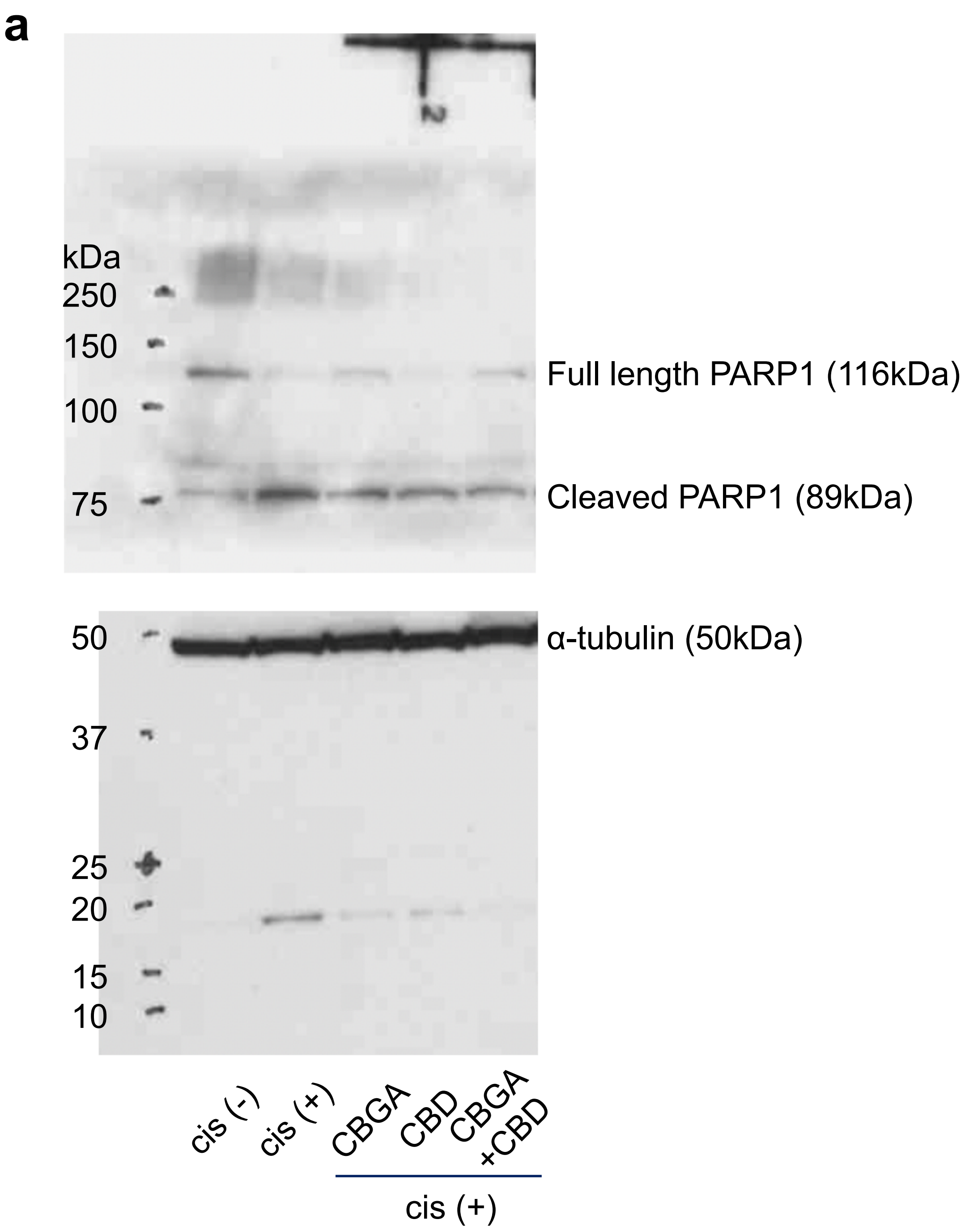

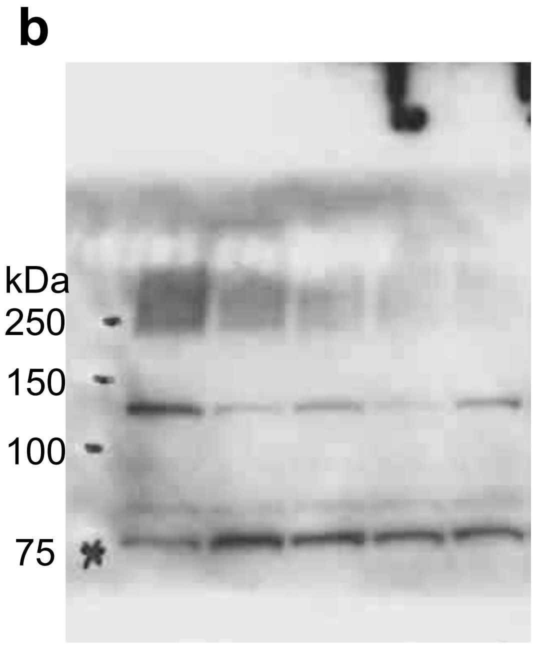

Fig. S2. Full image of western blotting in Fig. 3d, PARP expression in kidneys from cisplatin (-) and cisplatin-treated mice with CBGA, CBD and CBGA+CBD. (**a**) short and (**b**) long exposures.

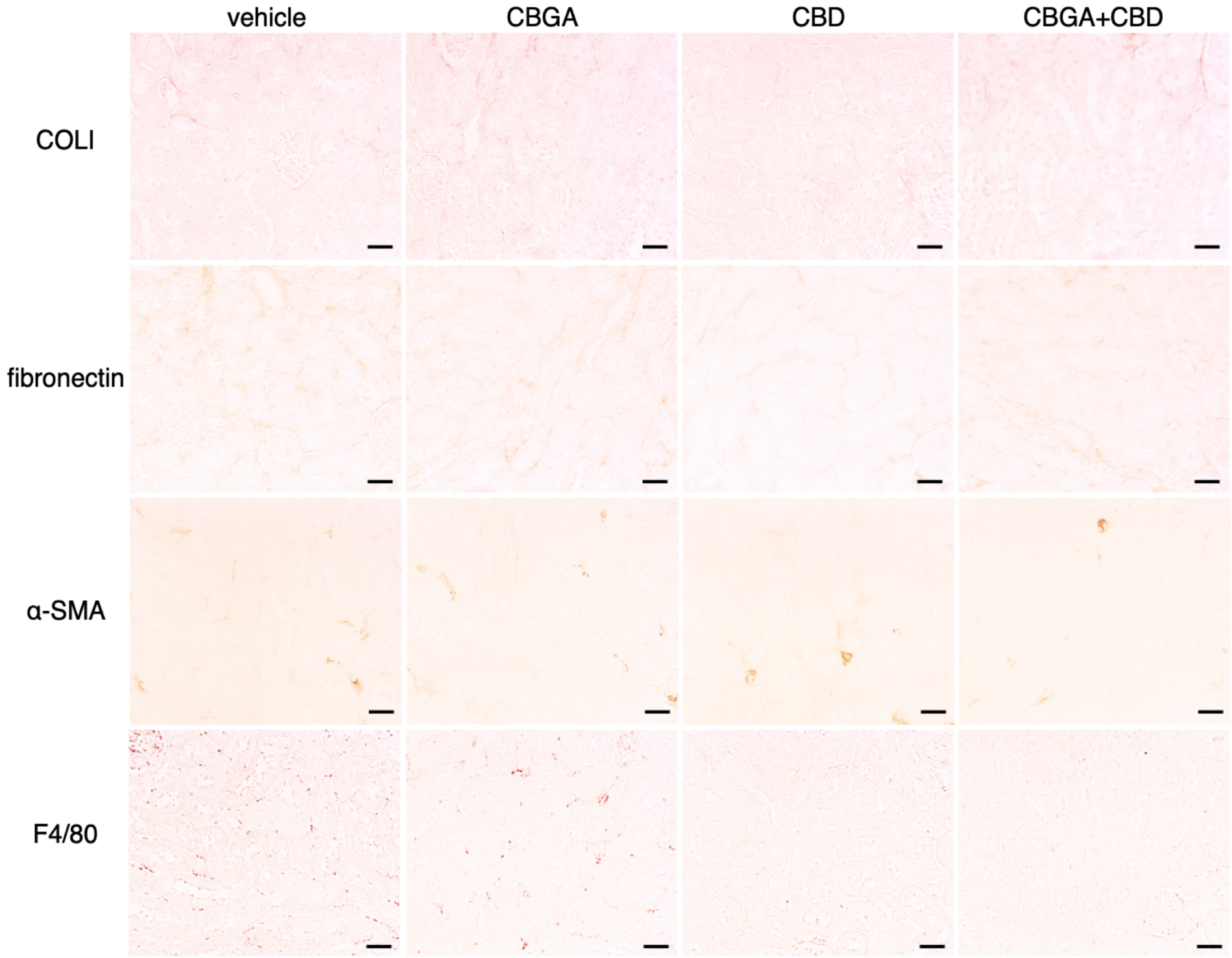

Fig. S3 Representative images of immunostainings for collagen type I, fibronectin, α-SMA and F4/80 from CLK kidneys treated with vehicle, CBGA, CBD and CBGA+CBD (magnification x200). Scale bars represent 50 μm.

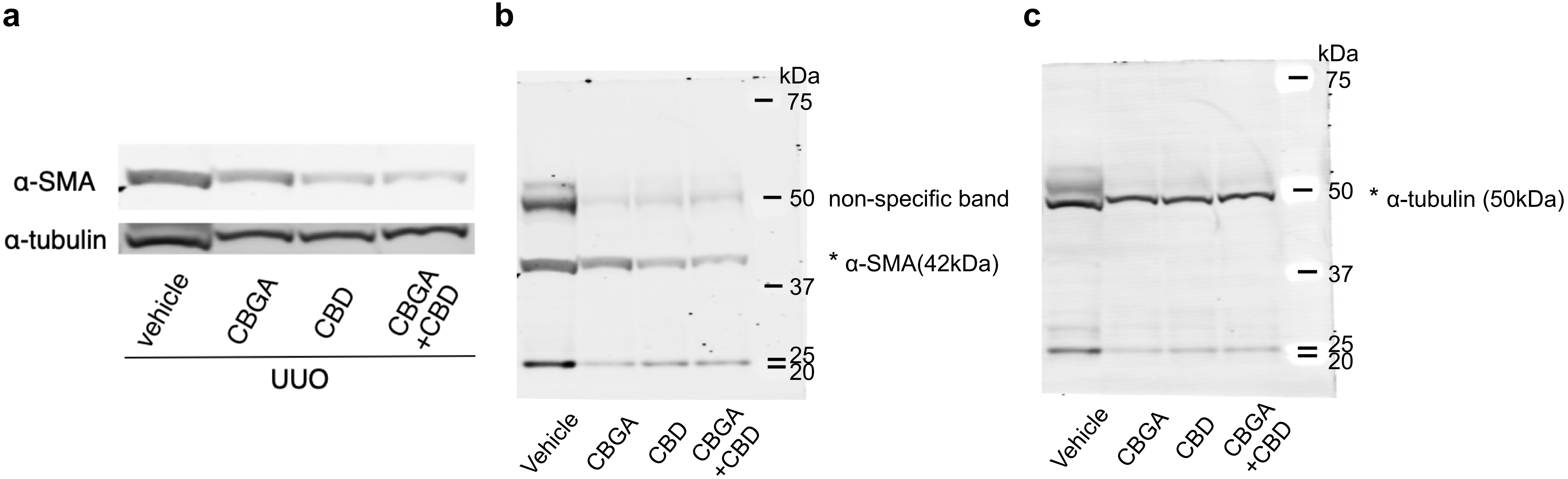

Fig. S4. (**a**) Composite image of western blotting of α-SMA protein expression in cortical UUO kidney tissue from mice treated with vehicle, CBGA, CBD and CBGA+CBD. (**b, c**) Full images of western blotting with anti-α-SMA (b) and anti-α-tubulin (c) that were used to assemble composite panel (a).

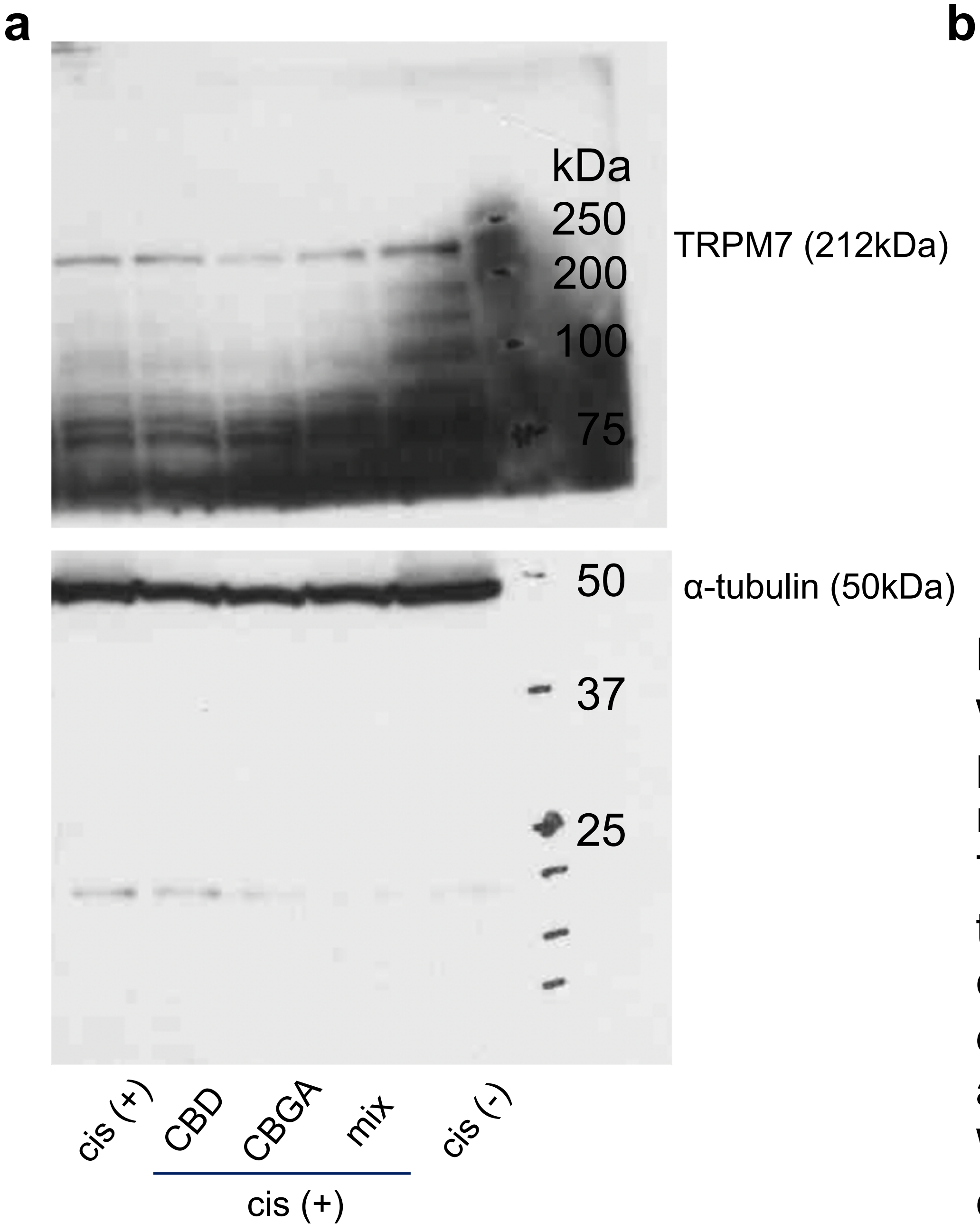

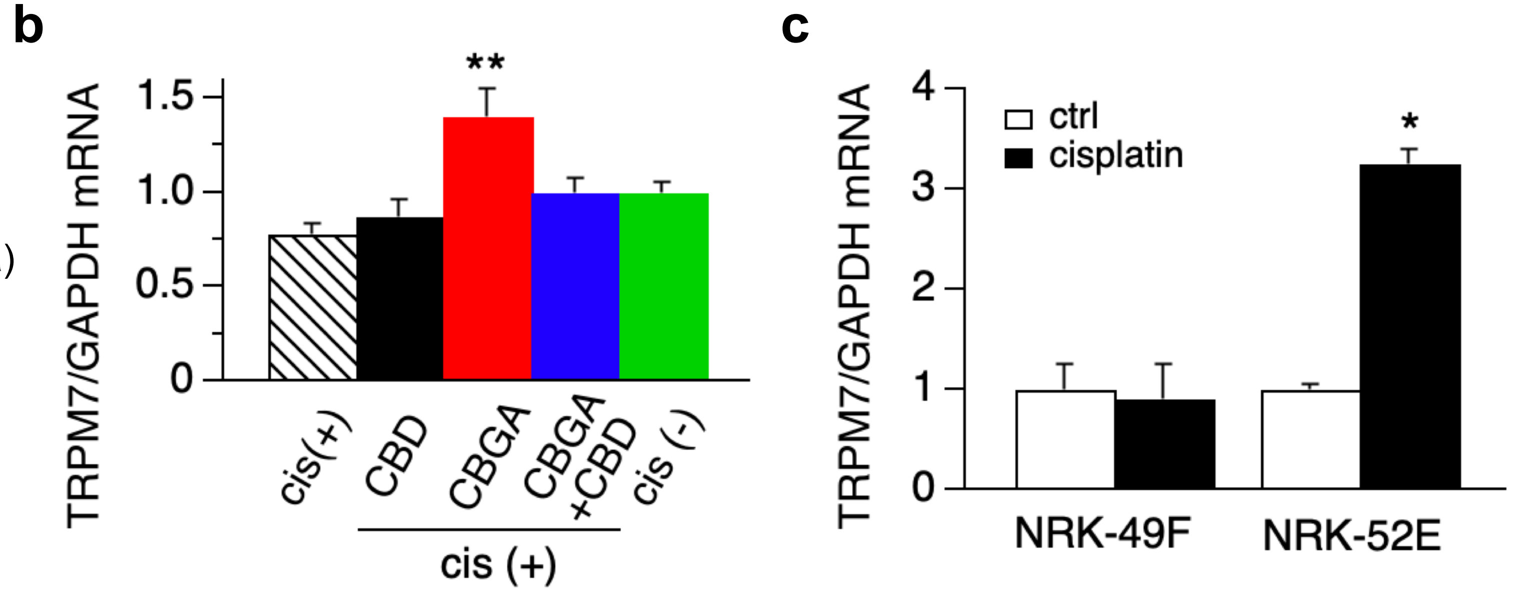

Fig. S5. The levels of kidney TRPM7 expression. (**a**) Full images of western blotting shown in **Fig. 7d.** Expression of TRPM7 (upper panel) and α-tubulin (lower panel) in kidneys from cisplatin-treated mice with CBD, CBGA and CBGA+CBD or cisplatin (-) control. (**b**) The level of TRPM7 mRNA expression in kidneys from cisplatin-treated mice with CBD, CBGA and CBGA+CBD or cisplatin (-) control. **P<0.01 vs. cisplatin (+). (**c**) The level of TRPM7 mRNA expression in normal rat kidney fibroblast cell line (NRK-49F, left) and epithelial cell line (NRK-52E, right) treated with 20 μM cisplatin. White bars are control, black bars are cisplatin treatment. *P<0.05 vs control.

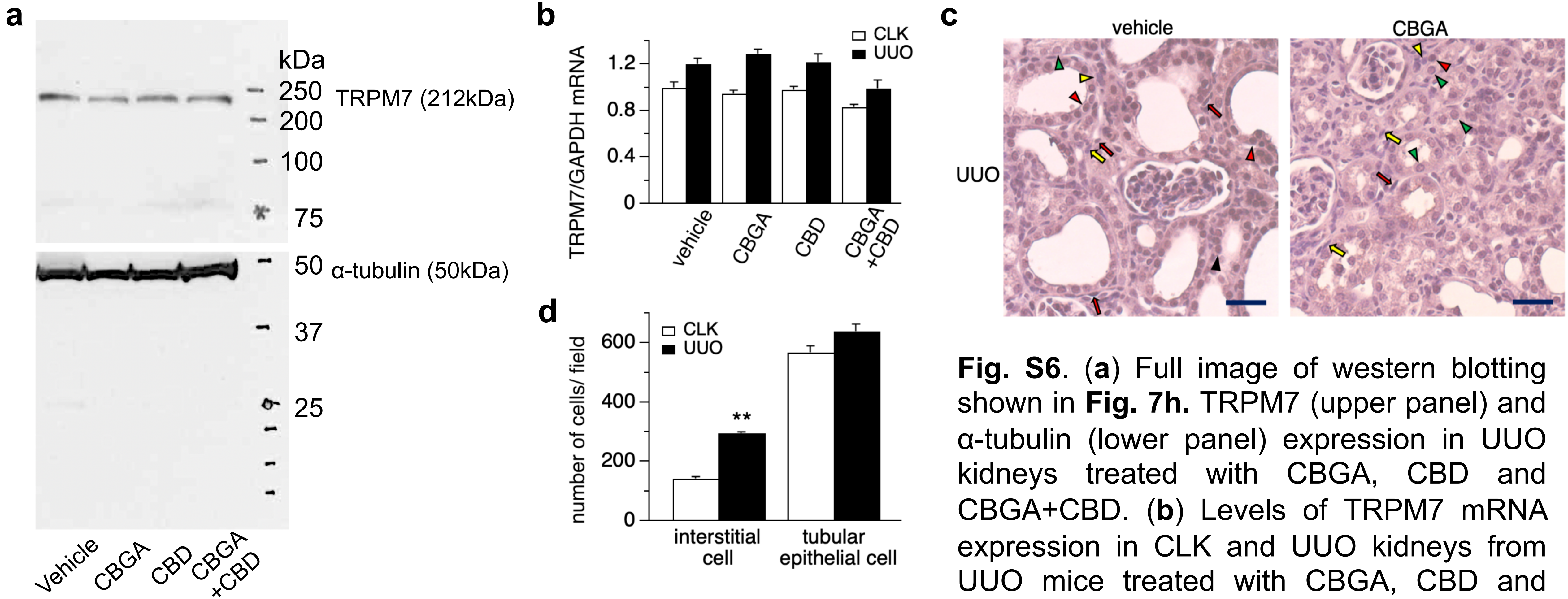

Fig. S6. (**a**) Full image of western blotting shown in **Fig. 7h.** TRPM7 (upper panel) and α-tubulin (lower panel) expression in UUO kidneys treated with CBGA, CBD and CBGA+CBD. (**b**) Levels of TRPM7 mRNA expression in CLK and UUO kidneys from UUO mice treated with CBGA, CBD and CBGA+CBD. (**c**) Representative images of immunostaining of TRPM7 (magnification x400) in UUO kidneys from UUO mice treated with vehicle (left panel) or CBGA (right panel). Scale bars represent 50 μm. Green arrow heads indicate TRPM7-low expression and red arrow heads are high expression levels of TRPM7, yellow arrow heads indicate TRPM7-negative (TRPM7 staining not detectable) in renal tubular epithelial cells. Yellow arrows point to TRPM7-negative and red arrows point to TRPM7-positive renal interstitial cells. (**d**) The number of interstitial and tubular epithelial cells in CLK and UUO kidneys from UUO kidneys treated with vehicle.

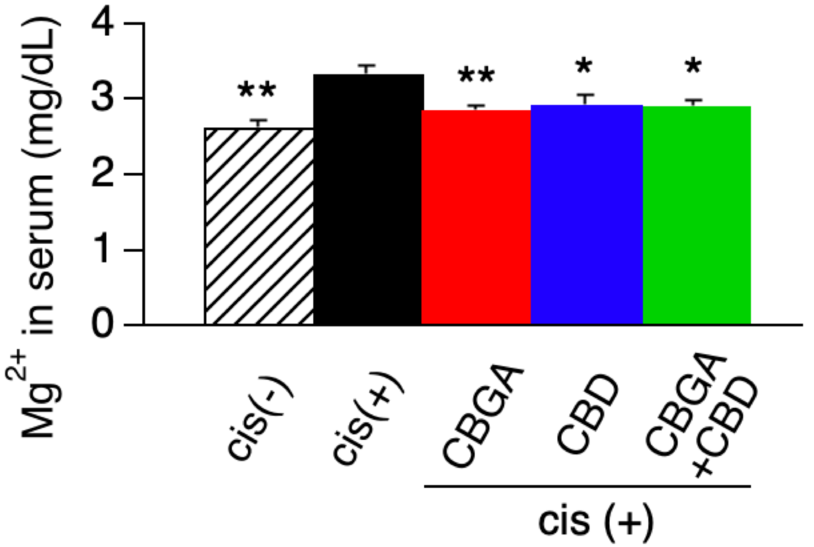

Fig. S7 Magnesium level in serum was measured at day 3 using Magnesium assay kit (BioAssay System, USA). Magnesium concentration in serum increased in cisplatin administered mice (black bar), it was suppressed by CBGA, CBD and CBGA+CBD treatment (red, blue, and green bars, respectively). *P<0.05, **P<0.01 vs cisplatin (+)

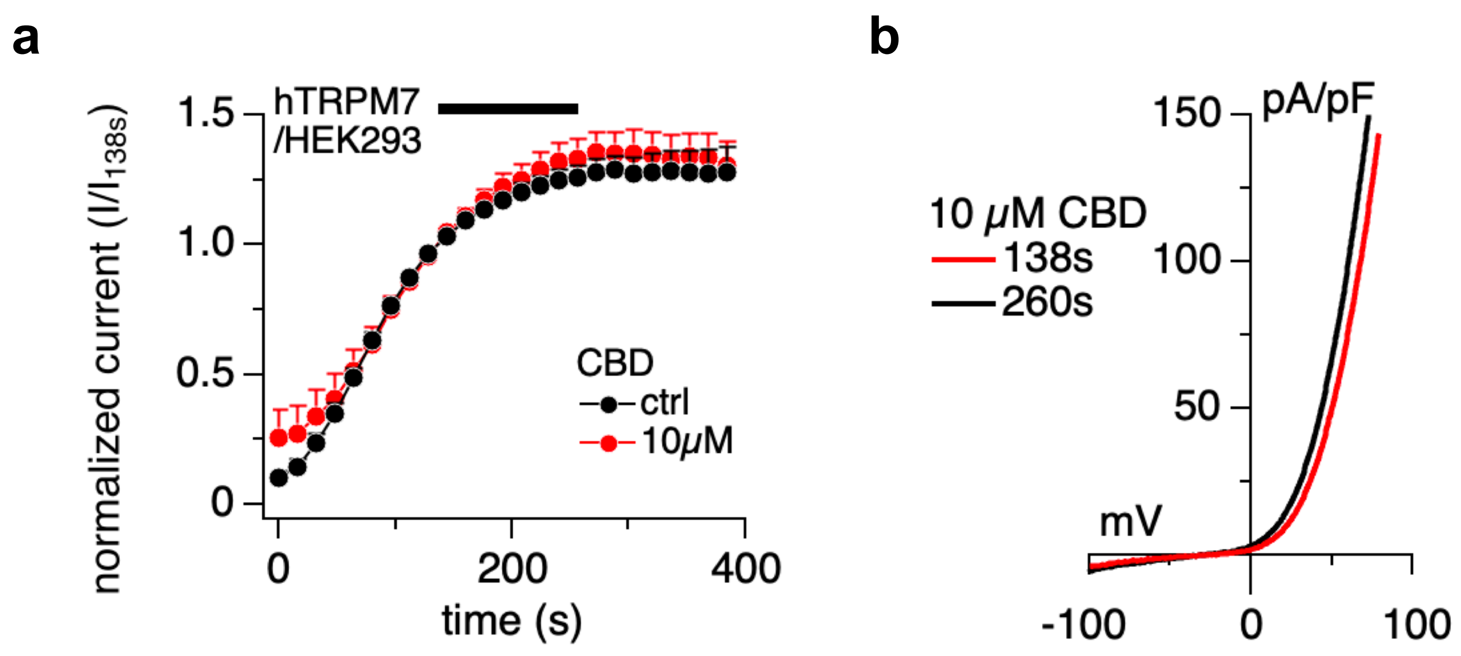

Fig. S8 CBD did not suppress TRPM7 channels. (**a**) Effect of CBD on TRPM7 currents in HEK293-TREx cells over-expressing human TRPM7. Average TRPM7-mediated outward currents at +80 mV extracted from ramp currents delivered at 0.5 Hz and plotted as a function of time. 10 μM CBD (red circles, n=6) was applied from 140 s to 260 s (black bar). Standard Ringer with just vehicle acetonitrile was used as control (black circles, n=6). (**b**) Average I/V curve of TRPM7 currents extracted before (138 s, black line) and after (260 s, red line) 10 μM CBD application.
